# Single-cell spatial analysis with Xenium reveals anti-tumour responses of *CXCL13* + T and *CXCL9*+ cells after radiotherapy combined with anti-PD-L1 therapy

**DOI:** 10.1038/s41416-025-03088-0

**Published:** 2025-07-16

**Authors:** Shunsuke A. Sakai, Hidekazu Oyoshi, Masaki Nakamura, Tetsuro Taki, Kotaro Nomura, Hidehiro Hojo, Hidenari Hirata, Atsushi Motegi, Yuka Nakamura, Junko Zenkoh, Keiju Aokage, Akira Hamada, Motohiro Kojima, Takeshi Kuwata, Katsuya Tsuchihara, Tetsuo Akimoto, Junichi Soh, Tetsuya Mitsudomi, Masahiro Tsuboi, Genichiro Ishii, Yutaka Suzuki, Ayako Suzuki, Riu Yamashita, Shun-Ichiro Kageyama

**Affiliations:** 1 Division of Translational Informatics, Exploratory Oncology Research and Clinical Trial Center, National Cancer Center, Kashiwa, Chiba, 277-8577 Japan; 2 Department of Integrated Biosciences, Graduate School of Frontier Sciences, The University of Tokyo, Chiba, 277-8568 Japan; 3 Department of Radiation Oncology, National Cancer Center Hospital East, Kashiwa, Chiba, 277-8577 Japan; 4 Division of Radiation Oncology and Particle Therapy, Exploratory Oncology Research and Clinical Trial Center, National Cancer Center, Kashiwa, Chiba, 277-8577 Japan; 5 Department of Pathology and Clinical Laboratories, National Cancer Center Hospital East. Kashiwa, Chiba, 277-8577 Japan; 6 Department of Thoracic Surgery and Oncology, National Cancer Center Hospital East, Kashiwa, Chiba, 277-8577 Japan; 7 Pathology Division, Exploratory Oncology Research & Clinical Trial Center, National Cancer Center, Kashiwa, Chiba, 277-8577 Japan; 8 Department of Computational Biology and Medical Sciences, Graduate School of Frontier Sciences, The University of Tokyo, Chiba, 277-8562 Japan; 9 Division of Thoracic Surgery, Department of Surgery, Kindai University Faculty of Medicine, Osaka-Sayama, Japan; 10 Department of Surgical Pathology, Kyoto Prefectural University of Medicine, Graduate School of Medical Science, Kyoto, Japan; 11 Department of Genetic Medicine and Services, National Cancer Center Hospital East, Kashiwa, Chiba, 277-8577 Japan

**Keywords:** Cancer immunotherapy, Tumour biomarkers, Non-small-cell lung cancer, Radiotherapy

## Abstract

**Background:**

The standard treatment for unresectable non-small cell lung cancer (NSCLC) is anti-PD-L1 therapy combined with chemoradiotherapy (anti-PD-L1-CRT). Although some patients achieve complete cancer eradication and cure, more than half of patients retain persistent cancer cells. Our research aimed to unravel the nuanced mechanisms involved in both immune attack and evasion induced by anti-PD-L1-CRT with single cell spatial transcriptome.

**Methods:**

Xenium is a cutting-edge single-cell spatial analysis tool that enables pathology-based and single-cell analyses while preserving spatial information. In our study, we used Xenium to identify the tumour microenvironment (TME), immune dynamics, and residual cancer cells at the single-cell level following treatment with anti-PD-L1-CRT.

**Results:**

Posttreatment alterations included a significant increase in *CXCL9*+ cells and *CXCL13* + T cells, particularly around tumour cells. Additionally, we discovered that *CXCL13* + T cells directly impact cancer cells in the posttreatment environment. Moreover, we identified clusters of immune-cold cancer cells posttreatment, revealing their activation of DNA repair pathways and high proliferative capacity. The novel spatial analysis tool Xenium enabled identification of the immune environment at the single-cell level following treatment with anti-PD-L1-CRT, elucidating its characteristics.

**Conclusions:**

These findings suggest potential advancements in developing new treatments to improve posttreatment immune responses and address resistance challenges.

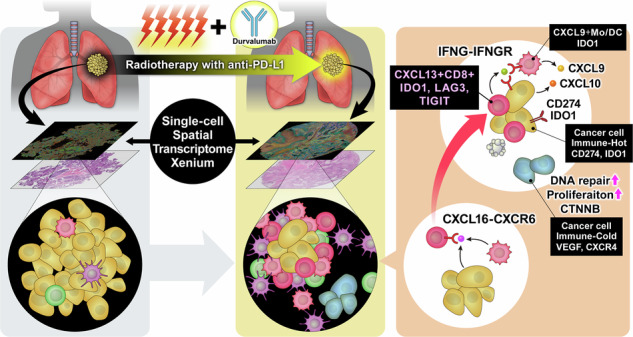

## Introduction

Radiotherapy (RT) is an important component of cancer treatment and is administered to approximately 50–60% of all cancer patients. For the past century, the major antitumour effect of RT has been thought to occur through induction of lethal DNA damage. Recently, accumulating evidence has revealed that immune responses and immunological cell death also contribute to the antitumour effect of RT [1, 2]. Exploitation of the immune response to improve cancer treatment has led to development of immune checkpoint inhibitors (ICIs), and many clinical trials of RT and chemoradiotherapy (CRT) combined with ICIs have been undertaken. Phase I and II trials of anti-PD-1 or anti-PD-L1 therapy combined with CRT have shown promising responses in some cancer types [3–5]. Notably, addition of anti-PD-L1 therapy significantly improved the effect of CRT on non-small cell lung cancer (NSCLC) in a phase III study [6, 7]. Furthermore, phase I and II trials of anti-PD-1 or anti-PD-L1 therapy combined with CRT have shown promising responses in other cancer types, such as head and neck, pancreatic and colorectal cancer [3–5]. However, several trials of anti-PD-1/PD-L1 combined with CRT have reported negative or inconclusive results [8–13], and that concurrent anti-PD-L1 therapy does not provide a survival benefit [14]. Furthermore, more than half of patients experience recurrence in NSCLC patients despite adjuvant anti-PD-L1 therapy [8]. These findings suggest the inadequate effect of the simple combination of anti-PD-1/PD-L1 therapy and CRT, and it is necessary to optimize ICI therapy combined with CRT. Moreover, the underlying mechanisms involved after treatment with an anti-PD-1/PD-L1 antibody combined with CRT need to be determined to establish a diagnostic tool for biomarker selection and/or integrate therapies targeting additional ICI targets.

Reports on immune environment changes after CRT with anti-PD-L1 therapy in patient tissues are still limited. Recent spatial analyses in esophageal cancer have shown infiltration of PD-L1 and IDO1-expressing cells post-treatment [15]. However, many findings on post-radiotherapy changes rely on preclinical models, and definitive conclusions remain elusive [16–23]. In contrast, immune changes following anti-PD-1/PD-L1 therapy have been extensively analyzed in patient tissues, highlighting the roles of regulatory T cells and CXCL13 + T cells, though their precise localization and function within the tumor microenvironment (TME) remain unclear [24–29].

Xenium, a novel technology allowing for multi-in situ hybridization, preserves spatial information for single-cell analysis [30, 31], enabling integrated analysis with haematoxylin and eosin (HE) staining and straightforward identification of treatment-responsive or treatment-resistant areas.

In this study, we performed novel single-cell spatial analysis using tissues following treatment with concurrent CRT and anti-PD-L1 therapy (anti-PD-L1-CRT) for the identification of treatment-induced changes in the immune environment. Initially, we performed technical and histological assessment using Xenium on surgically resected specimens following anti-PD-L1-CRT. Subsequently, we aimed to elucidate alterations in the immune environment after anti-PD-L1-CRT and to unravel the mechanisms driving treatment efficacy and resistance.

## Methods

### Tissue

A total of 12 biopsies of resected specimens were collected from 8 patients (surgery alone [32]: 1, preoperative CRT: 2, preoperative ICI-CRT: 5). Biopsy or surgical specimens were taken from previous patients [33]. This study was approved by the National Comprehensive Cancer Study Board (NCCHE) Institutional Review Board (Protocol Number 2022-407). Preoperative CRT was performed by platinum-based chemotherapy combined with RT (45 Gy in 25 fractions), and preoperative ICI-RT was performed according to the predefined protocols [33] (https://jrct.niph.go.jp/en-latest-detail/jRCT2080224981), carboplatin, paclitaxel and durvalumab concurrent with RT (50 Gy in 25 fractions). All patients who received preoperative CRT/ICI-CRT were at UICC 8th Stage IIIA.

### Experimental protocol for Xenium analysis

In situ expression analysis with Xenium was performed using Xenium Slides & Sample Prep Reagents (PN-1000460, 10x Genomics). FFPE tissue blocks were sectioned at 5 μm thickness and placed on a Xenium slide (10x Genomics) according to the manufacturer’s protocol (CG000578, Rev A, 10x Genomics). Deparaffinization and decrosslinking were performed according to the manufacturer’s protocol (CG000580, Rev A, 10x Genomics). Probe hybridization, ligation and amplification were also performed according to the Xenium In Situ Gene Expression user guide (CG000582, Rev A, 10x Genomics). After that, autofluorescence quenching and nuclear staining were performed, and the slides were kept in the dark. The Xenium Analyser (10x Genomics) was run for the prepared Xenium slides. The Lung panel was kindly provided by Ayako S. The Immune panel was created using custom-ordered components [34]. The genes in the panels are listed in Supplementary Tables S1 and S2.

### Cell count analysis using Xenium data

The Python library stLearn (v0.4.12) [35] was used to extract information on gene expression per cell and area per cell from Xenium In situ data. We compared cell counts for n ≥ 1 and n ≥ 2 and concluded that n ≥ 2 allows for a more specific evaluation of cell numbers. Positive (n ≥ 2) and negative (n = 0) results were defined for each cell for the 302 genes in the panel for each sample.

### Gene expression analysis

#### Tumour regions

The tumour region was delineated based on expression of *KRT* and *CDH1*, confirmed through HE staining patterns, and validated by a pathologist. Gene expression was assessed using gene density (gene expression/area) calculated by Xenium Explorer ver. 1.2. The analysis was conducted over the entire field.

### Field analysis

The delineated tumour was subdivided into distances of 20 μm, 70 μm, and 120 μm and segmented using Xenium Explorer, after which the gene density was analysed in each region. Clustering was performed by unweighted pair-group using centroids (UPGMC) using the Euclidian distance. The analysis was conducted only for Patient 7, which exhibited sufficient stromal content and allowed for distance analysis. The results represent the cumulative values from three locations.

### Calculation of the distance from the tumour surface

The “tl.cci.grid” function of stLearn was used for gridding Xenium data at 10 μm intervals in the posttreatment sample of Patient 7. To extract grids containing tumour tissue, *CDH1* + , *SFTPB* + , *SFTPC* + , and *SFTPD*− grids were extracted. Next, automatic contour extraction was performed using the Python library opencv (v4.8.1) to delineate the tumour surface based on grids containing the tumour. Specifically, median filtering (3 * 3 kernel) was performed using the “medianBlur” function, followed by smoothing (3 * 3 kernel) using the “blur” function to clarify the tumour boundaries. The “findContours” function was subsequently used to contour and extract only the first contour in the hierarchy.

Finally, to calculate the distance of each grid from the tumour surface, the grids were transformed into a graph structure (weighted by Euclidean distance), and the “multi_source_dijkstra” function in the Python library network (v3.1) was used to calculate the shortest distance from the grids on the tumour surface to the other grids.

### Hot and cold comparison analysis

Comparison of hot and cold regions was conducted at both the single-cell and region levels. For Patient 7, cancer cell regions were segmented at the single-cell level using Xenium Explorer. Segmentation was verified using HE staining, and poorly segmented cells were excluded. Segmentation was defined as either hot (*CD274* + ) or cold (*CD274* − ) cells. For region-level analysis, areas with high *CD274* expression were manually delineated. The validity of each cell and region was confirmed by checking *CD274* expression using Python. Hot/Cold cells consisted of 100/74 cells in Patient 7 and 35/101 in Patient 12, and the regions were defined using five distinct areas for each.

### Pathway enrichment analysis

Samples from the custom panel involving Patient 7 were used; the ‘pp.filter_genes’ and ‘pp.filter_cells’ functions in the Python library scanpy (v 1.9.3) (Wolf et al. Genome Biology, 2018) were used to filter out genes with less than 10 total counts and cells with fewer than 10 counts of mRNA in the cell. Scanpy’s ‘pp.normalize_total’ function was used to determine gene expression levels. The “tl.cci.grid” function in stLearn (v0.4.12) was subsequently used to grid the entire field of view into 125*125 = 15625 spots, and genes with expression levels in the top 5% of all these spots were defined as DEGs. GO enrichment analysis was then performed using the ‘enrichr’ function of the Python library gseapy (v1.0.6), with a background of 302 genes in the whole panel of Xenium In situ, for genes with differential expression in each spot.

### Comparative analysis

Welch’s t test was used for significance difference tests (RRID:SCR_008058); the Benjamini–Hochberg method was used to correct for multiple testing; Cohen’s *d* was used for effect size; the effect size was calculated using a standardized pair group using the centroid method.

### Gene expression analysis of CXCL9+ and CXCL13+ cells

To identify cell types among *CXCL13*+ and *CXCL9*+ cells, lymphocyte markers (*CD3E*, *CD4*, *CD8A*, *GZMB*, and *CD19*), macrophage markers (*MSR1*, *CD68*, and *CD163*), dendritic cell markers (*MS4A4A*, *ITGAX*, and *HLA-DQB2*), epithelial markers (*KRT8* and *CDH1*) and ICI targets (*CD274*, *CTLA4*, *IDO1*, *LAG3*, and *TIGIT*) were used. For each marker, the *z* scores of average expression in *CXCL13*+ and *CXCL9*+ cells were calculated.

Similarly, to determine the role of *CXCL13* − *CD8* + , CXCL13 − *CD8* + , *CXCL9* − *CD68* + , *CXCL9* + *CD68* + , *CD274* − *EPCAM*+ and *CD274* + *EPCAM*+ cells in the immune response, receptor genes (*CCR1*, *CCR2*, *CCR3*, *CCR4*, *CCR6*, *CCR7*, *CCR8*, *CXCR2*, *CXCR3*, *CXCR4*, *CXCR6*, *IL10RB*, *IL12RB1*, *IL23R*, *IL3RA*, *IL6R*, *IL7R*, *IFNAR1*, *IFNAR2*, *IFNGR1*, *IFNGR2*, and *IFNLR1*), humoral immune genes (*CCL2*, *CCL3*, *CCL3L1*, *CXCL9*, *CXCL10*, *CXCL13*, *CXCL16*, *IFNG*, *IL18*, and *IL33*), regulator genes (*TLR2*, *TLR4*, *TMEM173*, *CGAS*, *DDX58*, *IRF1*, *IRF9*, *NFKB1*, *NFKB1*, *STAT1*, and *CIITA*), the percentage of positive cells and the z score of the average expression levels were calculated.

### Availability of data and materials

The clinical specimen data, including Xenium data produced during this research, are archived in the DDBJ BioProject. The accession number is currently in the process of being requested. All the data necessary for assessing the conclusions presented in the paper can be found within the paper itself and/or in the Supplementary Materials.

All the code used for the analysis has been uploaded to the web server of the Human Genome Center, University of Tokyo (https://www.hgc.jp/~ssakai/publication/Lung/code.zip).

## Results

### Study objectives and design

**Fig. 1:**
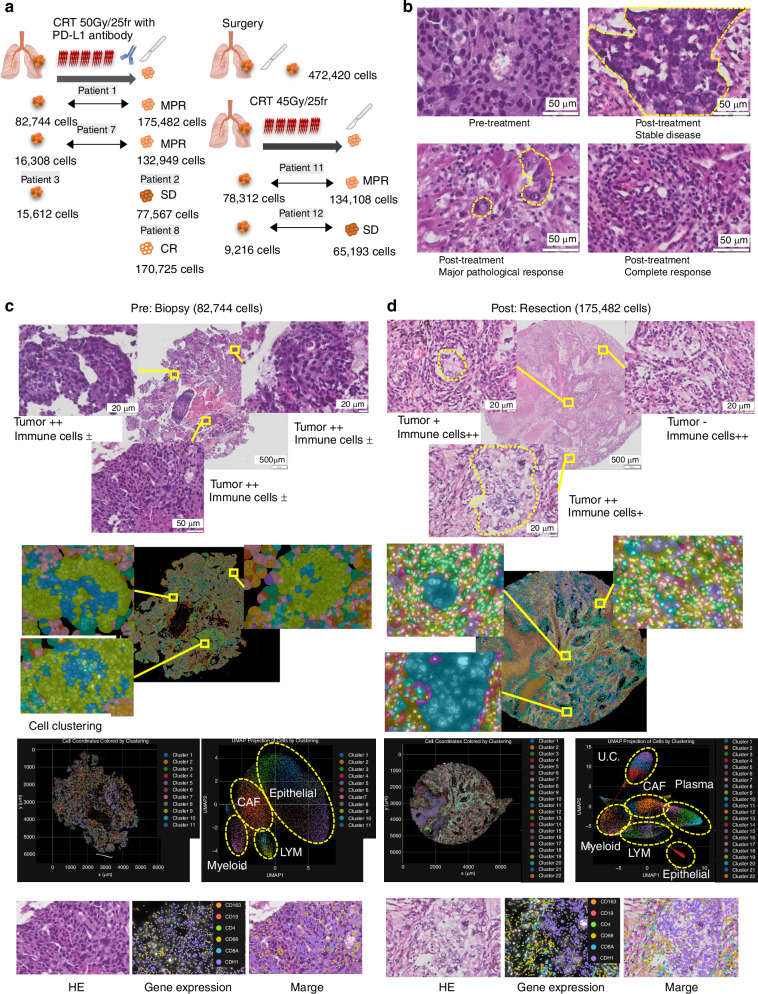
Evaluation of tissues and Xenium analysis after treatment with anti-PD-L1 combined with chemoradiotherapy. **a** Characteristics of the tissue samples used in this study. The cell number indicates the number of cells that can be analysed with Xenium. Posttreatment histology revealed stable disease (SD), major pathological response (MPR), and complete response (CR). **b** Representative HE staining image of pre- and posttreatment tumours. Cancer cells were delineated via HE staining (yellow line). **c**, **d** Representative images of HE staining, cell clustering and gene expression. After each cell line was distinguished and utilizing 302 gene expression patterns, pretreatment cells were categorized into 11 clusters, and posttreatment cells were classified into 22 clusters. Each cluster was classified as epithelial (*KRT5* + , *CDH1* + ), CAF (*COL1A1* + ), myeloid (*CD68* + , *CD163* + ), LYM (lymphocyte, *CD3E* + , *CD4* + , *CD8* + ), plasma (*POU2AF1* + ), or U.C. (dead cells or unclassified cells).

The aim of this study was to elucidate alterations in the immune environment after treatment with anti-PD-L1-CRT and to investigate the underlying mechanisms at the single-cell level via use of Xenium. First, we evaluated tissue conditions using HE staining and performed technical evaluation of the results by comparing marker gene expression via HE staining. The study included two pairs of tissue specimens isolated before and after treatment with anti-PD-L1-CRT. In addition, the analysis included one patient with pretreatment only and two patients with posttreatment only. In these patients, the pretreatment tissue quantity was limited, and an adequate number of cells could not be obtained with Xenium. The primary focus of this study was pre-post analysis of anti-PD-L1-CRT. However, for comparative purposes, the analysis also included two patients with pre-post pairs of CRT alone and one patient with resection alone (Fig. 1a and Tables 1, 2).

**Table 1:** Patient characteristics.

Patients	T stage	N stage	cStage	Tissue type^*^	EGFR	ALK	PD-L1
			(UICC 8th)				IHC
Patient 1	2b	2	3 A	LUAD	Wild	Wild	10-24%
Patient 2	2a	2	3 A	LUAD	Exon19 del	N.D.	<1%
Patient 3	2a	2	3 A	LUAD	Wild	N.D.	N.D.
Patient 7	2a	2	3 A	LUAD	Wild	N.D.	1-4%
Patient 8	2a	2	3 A	LUAD	Wild	N.D.	1-4%
Patient 11	1c	2	3 A	LUAD	Exon19 del	Wild	N.D.
Patient 12	3	1	3 A	LUAD	Wild	Wild	25-49%
Resection alone	1c	1	2B	LUAD	Wild	Wild	N.D.

^*^LUAD Lung adenocarcinoma.

**Table 2:** Treatment of each patient.

Patients	Response^*^	Chemotherapy regimen^**^	Radiation Dose
Patient 1	MPR	CBCDA + PTX+Durva	50 Gy/25 fr
Patient 2	SD	CBCDA + PTX+Durva	50 Gy/25 fr
Patient 3	CR	CBCDA + PTX+Durva	50 Gy/25 fr
Patient 7	MPR	CBCDA + PTX+Durva	50 Gy/25 fr
Patient 8	CR	CBCDA + PTX+Durva	50 Gy/2 5fr
Patient 11	SD	CDDP + PEM	45 Gy/25 fr
Patient 12	MPR	CDDP + VNR	45 Gy/25 fr
Resection alone	N.D.	N.D.	N.D.

^*^MPR major pathological response, *SD* stable disease, *CR* complete response, *PR* partial response, ^**^CBCDA carboplatin, *PTX* paclitaxel, *Durva* durvalumab, *CDDP* cisplatin, *PEM* pemetrexed, *VNR* vinorelbine. Durvalumab was concurrently administered with PTX, CBCDA and Radiotherapy.

### Tissue quality assessment and Xenium validation

Initially, we conducted material evaluation of posttreatment tissues, with HE staining and Xenium staining for whole biopsy tissues and 5 mm core samples of resected tissues, respectively. The cell count for each tissue was assessed using Xenium Explorer, confirming an ample quantity of cells. As part of the standard pathological diagnostic process, pathologists identify cancer cells and immune cells by evaluating the morphology and size of nuclei and cells through HE staining, thereby assessing the efficacy of treatment. In pretreatment tissue, we found densely packed cancer cell clusters with relatively uniform nuclei and cytoplasm (Fig. 1b and Supplementary Figs. S1A-G). In contrast, in posttreatment tissues, we observed increased immune cell infiltration into tumours and diverse tissue patterns, such as regions where cancer cells were attacked by immune cells, areas where cancer cells were replaced by immune cells, and sites where viable cancer cells coexisted with immune cells (Fig. 1b and Supplementary Figs. S1A-F).

Next, to evaluate Xenium and ensure that we had enough cells, we performed Xenium analysis, which was used to assess expression of 302 genes (Table S1) in samples obtained pre- and post-PD-L1-CRT treatment. Sufficient cell count information was obtained for all samples (Fig. 1a). Based on the expression patterns of the 302 genes, each cell was clustered into 11 groups in the pretreatment phase and 22 groups in the posttreatment phase by Xenium Analyzer (10X Genomics) (Fig. 1c, d). The data obtained by gene expression and clustering analyses were merged with HE staining images using Xenium Explorer version 1.2. By using this approach, the cell types identified by HE staining and morphological classification were confirmed and shown to correspond to cells in which genetic markers of epithelial cells (*CDH1*), monocytes/macrophages (*CD68* and *CD163*), lymphocytes (*CD4* and *CD8*), and B lymphocytes (*CD19*) were expressed (Fig. 1c, d and Supplementary Fig. S1H). Additionally, we assessed tissue structures, including lymphatic vessels (*PDPN*), smooth muscle (*LMOD1*), and vascular endothelium (*PECAM1*), by comparing HE staining with marker gene expression.

Expression of each gene, the groups based on which it was expressed, and location of the genes obtained by these Xenium analyses were consistent with the HE staining findings (Supplementary Fig. S1H). In this manner, we showed that Xenium analysis following treatment with anti-PD-L1-CRT yielded both adequate material quality and technical reliability, validating its suitability for our study. Moreover, we found that tumour cells and immune cells were morphologically altered by anti-PD-L1-CRT, and we next analysed gene expression in detail using Xenium.

### Characterization of cells and gene expression post-PD-L1-CRT

**Fig. 2:**
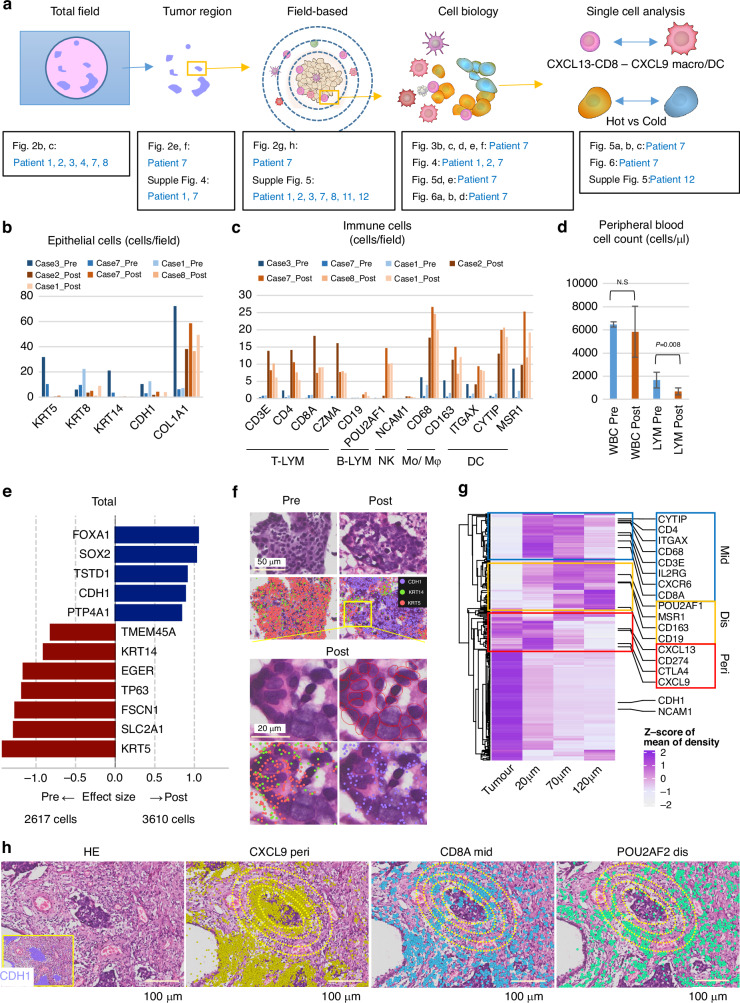
Characterization of gene expression profiles of cells in tissue samples obtained following combined treatment with anti-PD-L1 antibodies and chemoradiotherapy. **a** Workflow of the analysis in this study. **b**, **c** The number of cells in the total field analysed before (Patients 1, 2, and 7) and after (Patients 1, 3, 7 and 8) surgery. Positivity was defined as ≥2 or above. In all pre-post comparisons, a statistically significant difference was observed (P < 0.05, Student’s t-test). **d** The number of white blood cells (WBCs) and lymphocytes in the plasma. Pre: plasma within 2 weeks before treatment; Post: plasma within 2 weeks after resection. **e** Bar plot illustrating differences in the density of mRNA expression per cell area between pretreatment (Patient 1: 1029 cells, Patient 3: 1248 cells, and Patient 7: 340 cells) and posttreatment (Patient 1: 1502 cells, Patient 2: 1059 cells, and Patient 7: 1049). The x-axis indicates the effect size (Cohen’s d), and the y-axis indicates each gene with significant changes in expression (Welch’s t test, P < 0.05). **f** Confirmation of gene expression through Xenium Explorer. **g** Heatmap with dendrogram illustrating differences in the density of gene expression between manually delineated intertumoural, ≤20 μm, ≤70 μm, and ≤120 μm regions. The x-axis indicates each delineated region, and the y-axis indicates 302 genes in the Xenium I lung panel. **h** Merged images of gene expression and HE staining.

We analysed gene expression data obtained by Xenium using the following workflow (Fig. 2a). First, we systematically examined alterations following anti-PD-L1-CRT. We analysed the entire tissue (total field) and the delineated cancer cell region (field-based) separately and evaluated the alterations in cell and gene expression after treatment. Second, we examined the localization and function of cells through pathology-based analysis (“Cell Biology”), focusing in particular on histological images of immune cells interacting with cancer cells. Finally, we analysed *CXCL13* + CD8 T cells, which are effector cells, and immune-cold cancer cells on a cell-by-cell basis via “single-cell analysis”.

To investigate changes in the TME after treatment with anti-PD-L1-CRT, we first explored alterations in epithelial and immune cell numbers in the total field. We assessed cell type based on expression of marker genes: *KRT5*, *KRT8*, *KRT14*, and *CDH1* for epithelial cells; *COL1A1* for fibroblasts; and a panel of markers (T cells: *CD3E*, *CD4*, *CD8A*, *IL2RG*, and *CXCR6*; B cells: *CD19*, and *POU2AF1*; monocyte/macrophages: *CD68*, and *CD163*; and dendritic cells: *ITGAX*, *CYTIP*, and *MSR1*) for immune cells. We then compared the number of the cell counts per area for each type in tissue samples obtained pre- and posttreatment (n = 3 and 4, respectively) using *t* tests. We evaluated immune cells by HE staining and evaluated marker gene expression; cells with a score of 2 or higher were defined as positive (Supplementary Fig. S2). We observed a general decrease in epithelial cells after treatment and a particularly marked reduction in *KRT5*− and *KRT14*+ cells, which were almost undetectable in posttreatment samples (Fig. 2b). The remaining epithelial cells in posttreatment samples were largely *KRT8*+ and *CDH1*+ cells. While the number of *COL1A1*+ cancer-associated fibroblasts (CAFs) tended to increase in posttreatment samples, significant differences were not observed. The numbers of immune cells, including T lymphocytes (TLYMs), B lymphocytes (BLYMs), natural killer cells (NKs), monocytes/macrophages (Mo/Mφ), and dendritic cells (DCs), were significantly greater in posttreatment samples than in pretreatment samples (Fig. 2c).

We confirmed an increase in a broad range of immune cells, including lymphocytes, in tissues after CRT alone and anti-PD-L1-CRT (Fig. 2b, Supplementary Fig. S3). The increase in immune cells was particularly prominent in the anti-PD-L1 treatment group.　To confirm that these increases were not simply caused by an increase in white blood cells (WBCs), including lymphocytes (LYMs), in peripheral blood, we examined the WBC and LYM counts in the blood over a 12-week period from just before the initiation of treatment to surgery. Unlike within tissues after anti-PD-L1-CRT, WBC counts in peripheral blood decreased after the initiation of treatment compared to before the initiation of treatment (Fig. 2d). The observed discrepancy in lymphocyte counts between cancer tissue and peripheral blood posttreatment indicates that a specific and local immune response can be induced in cancer tissue.

To reveal changes in gene expression in the tumour region induced by anti-PD-L1-CRT, we manually delineated tumour regions via HE staining and compared gene expression levels via Xenium between pretreatment (Patients 1, 2, and 7) and posttreatment (Patients 1, 3, 7, and 8). Expression of *FOXA1*, *SOX2*, *TSTD1*, *CDH1*, and *PTP4A1* increased significantly (Welch’s t test, *P* < 0.05) in posttreatment, while that of *TMEM45A*, *KRT14*, *EGFR*, *TP63*, *FSCN1*, *SLC2A1*, and *KRT5* decreased significantly (*P* < 0.05) (Fig. 2e). Moreover, nuclear enlargement and chromatin condensation were observed posttreatment, along with obvious decreases in *KRT5* and *KRT14* and increases in *CDH1* expression (Fig. 2f). Taken together, these findings suggest that anti-PD-L1-CRT therapy dramatically alters expression of cytoskeletal genes, such as *KRT5* and *CDH1*, in tumours, along with changes in cell morphology.

As no common targetable pathways were identified in integrated analysis of pretreatment (3 patients) and posttreatment (4 patients), we conducted a targeted exploration in the pair analysis of Patient 1 and 7. These 2 patients presented with significant activation or inhibition of several genes; these genes included genes in targetable pathways such as *VEGFA*, *KIT*, *ERBB2*, and *EGFR* (Supplementary Fig. S4). According to our gene expression analysis focused on cancer tissues, activation of such targetable pathways is not common but rather individualized, and our findings suggest the need for individualized therapeutic strategies targeting treatments directed at these genes to discern the activated state following treatment with anti-PD-L1-CRT (Supplementary Fig. S5). Although treatment clearly changes cell numbers and gene expression, it is not known where these changes occur, i.e., whether specifically within the tumour or stroma. To clarify this point and screen more crucial cells and genes, spatial analysis was conducted for Patient 7, whose tissue had all the characteristics of posttreatment tissues (i.e., regions where cancer cells were attacked by immune cells, areas where cancer cells were replaced by immune cells, and sites where viable cancer cells coexisted with immune cells). Regions close to the tumour were segmented into the tumour and regions outside the tumour according to the distance from the tumour surface, namely, peritumour (peri, <20 μm), mid-peritumour (mid, 20-70 μm) and distal peritumour (distal, 70-120 μm), using Xenium Explorer; the cells within these regions and gene expression pattern were evaluated for clustering analysis. The peri cluster included immune-related genes such as *CXCL9* and *CXCL13*, the mid cluster included cytotoxic T lymphocyte (CTL) and macrophage markers such as *CD8* and *CD68*, and the distal cluster included B-cell markers and M2 macrophage markers such as *CD19* and *CD163* (Fig. 2g). The location of the genes was reconfirmed by Xenium Explorer analysis, and our analysis enabled us to distinguish the cells localized within or at the periphery of the tumour from those located further from the tumour (Fig. 2h). In our analysis of cancer cells, we found limited common upregulation of genes or pathways, making identification challenging. However, in our analysis of immune cells, we observed a broad increase in most cell types, posing difficulties in mechanism analysis. Through our spatial analysis, we identified *CXCL13* + , *CD274* + , *CTLA4*+ and *CXCL9*+ immune cells, which directly interact with the tumour, hypothesizing their pivotal role. Subsequent analyses were undertaken with a specific focus on this identified cluster.

### Single-cell spatial transcriptome analysis revealed the characteristics of *CXCL9*+ cells and *CXCL13*+ cells that accumulate near cancer cells after treatment with anti-PD-L1-CRT

**Fig. 3:**
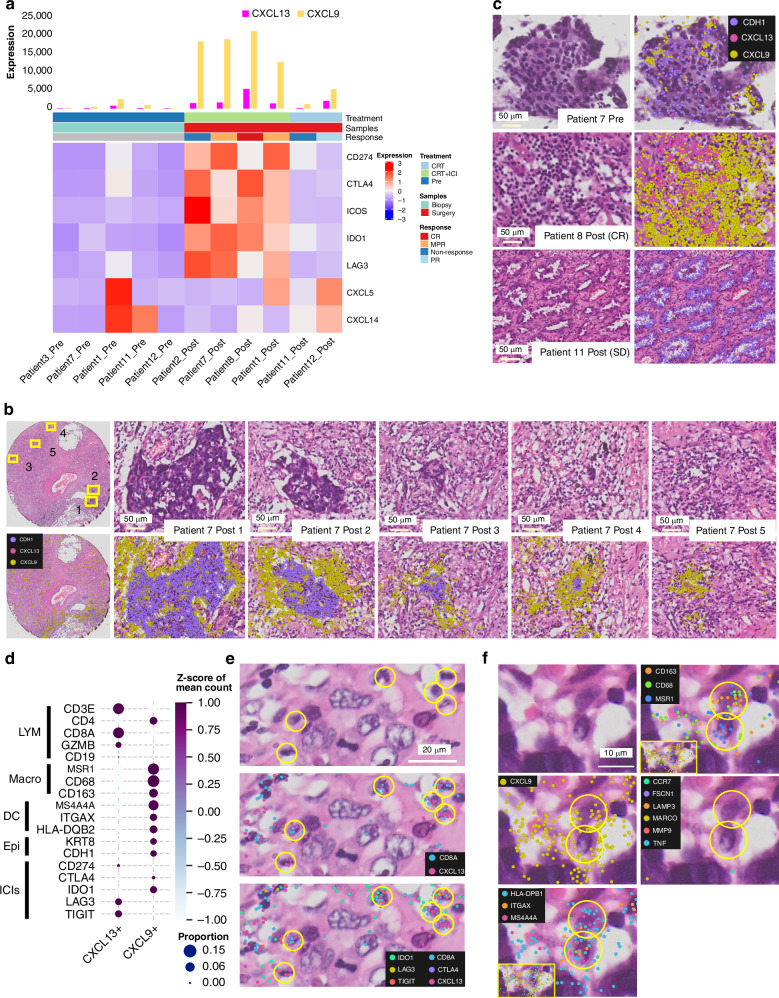
Single-cell spatial transcriptome analysis revealed characteristics of *CXCL9*+ and *CXCL13*+ cells that accumulated among cancer cells after anti-PD-L1 combination therapy. **a** Treatment, tissue, genotype, response, and number of cells expressing *CXCL9*, *CXCL13*, *CD274*, *CTLA4*, *ICOS*, *IDO1*, *LAG3*, *CXCL5*, and *CXCL14*. **b** Peripheral tumour gene expression of *CXCL13* and *CXCL9* in Patient 7. Posts 1-4 show residual cancer cells, and post 5 shows only accumulated lymphocytes. **c** *CXCL13* and *CXCL9* expression in Patient 7 pre, Patient 8 post, and Patient 11 posttissue. **d** Bubble chart with a colour scale showing the expression level and proportion of cell marker genes and ICI target genes in *CXCL13*+ and *CXCL9*+ cells using in situ Xenium data for Patient 7. The x-axis indicates the cells, the y-axis indicates each marker gene, the colour scale indicates the z score of the mean number of positive cells for each marker gene, and the circle size indicates the proportion of positive cells for each marker gene. **e** *CXCL13* + *CD8* + T cells coexpressing exhaustion marker genes on tumour cells. **f** *CD8* + T cells coexpressing exhaustion marker genes on tumour cells. **f** *CD68*− and *CD163*+ myeloid cells coexpressing *CXCL9*.

Spatial analysis of Patient 7 suggested interactions of *CD274* + , *CTLA4* + , *CXCL9* + , and *CXCL13+* cells with cancer cells. Recent reports have shown that *CXCL9+* and *CXCL13+* cells are strongly associated with the effect of anti-PD-1/PD-L1 therapy without RT [24–27]. We investigated *CXCL9+* and *CXCL13+* cells among all anti-PD-L1-CRT pre-post samples and CRT alone pre-post samples (Fig. 3a and Supplementary Fig. S5). As confirmed in Patient 7, upregulation of *CXCL9*+ and *CXCL13*+ cells and localization within the tumour were confirmed in all post anti-PD-L1-CRT tissues (Fig. 3a). In contrast, the number of *CXCL9*+ and *CXCL13*+ cells moderately increased in post-CRT alone tissue. In addition, we investigated expression of *CTLA4*, *ICOS*, *IDO1*, and *LAG3*, which are reported to be expressed in *CXCL13*+ cells, and the chemokines *CXCL5* and *CXCL14*. As for *CXCL13*+ cells, *CTLA4* + , *ICOS* + , *IDO1* + , and *LAG3*+ cells were increased in all tissues after anti-PD-L1-CRT, and no correlation with treatment was confirmed for *CXCL5* or *CXCL14* (Fig. 3a). In Patient 7, which exhibited high levels of cellular heterogeneity, large clusters of cancer cells were observed in some regions (Fig. 3b, Post 1, 2); in other areas, immune cell-mediated tumour elimination was observed (Post 3,4). Additionally, complete eradication of cancer cells was observed in some regions (Post 5). By using Xenium Explorer, we confirmed strong expression of *CXCL9* and *CXCL13* in almost all areas around the tumour, including at sites of tumour eradication (Fig. 3b). The same findings were obtained for another patient with complete response (Patient 8), while *CXCL9* and *CXCL13* expression was low in pretreatment tissues and tissues from patients after surgical resection alone (Fig. 3c), indicating that their expression was induced by anti-PD-L1-CRT.

Given that *CXCL9* and *CXCL13* expression appears to be induced by anti-PD-L1-CRT and that these cells localize to the vicinity of the tumour, we next sought to identify these cell types through the expression of genes that identify cell types. The analysis revealed that *CXCL13*+ cells were *CD8*+ lymphocytes and that *CXCL9*+ cells were double positive for macrophage markers (*MSR1*, *CD68*, and *CD163*) and DC markers (*MS4A4A*, *ITGAX*, and *HLA-DQB2*) (Fig. 3d). An analysis of all *CD8*+ cells revealed that *CXCL13* + *CD8+* cells accumulated inner- and peri-region of the tumour, indicating that they may directly interact with cancer cells. Moreover, these cells exhibited elevated expression levels of exhaustion markers, including TIGIT, LAG3, and IDO1. (Fig. 3e). While we did not observe an increase in expression of specific *CXCL9*+ cell markers, which was previously reported [28] after anti-PD-L1-CRT (Fig. 3f), we did observe double-positive macrophages/DCs, a characteristic feature of *CXCL9*+ cells localized around the tumour after anti-PD-L1-CRT (Fig. 3f). Moreover, we found that *CXCL9* was expressed not only by macrophages/DCs but also by some tumour cells and lymphocytes (Fig. 3d). Thus, we confirmed the increase in the number of *CXCL9*+ cells and *CXCL13*+ cells and their concentration within and peripheral to the tumour in all four patients following post-PD-L1-CRT. Additionally, we found that *CXCL13*+ cells were enriched in exhaustion marker-positive *CD8*+ lymphocytes.

### *CXCL13* + *CD8* + T cells accumulate in cancer tissue after treatment and are in close proximity to cancer cells

**Fig. 4:**
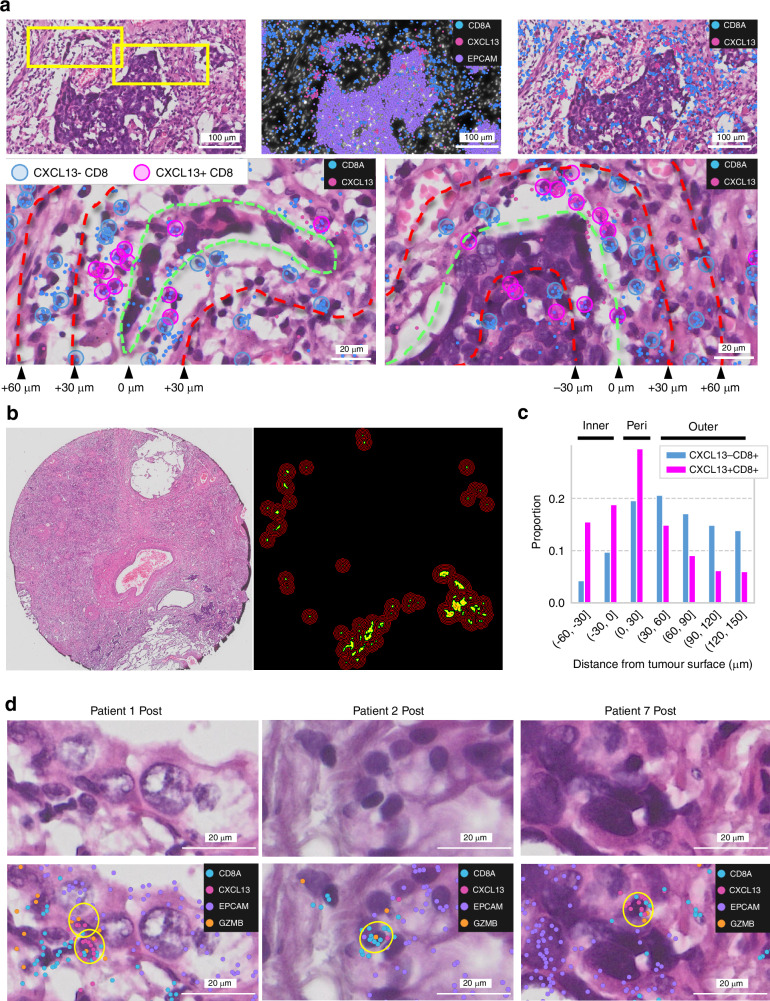
*CXCL13* + *CD8* + T cells accumulate in posttreatment cancer tissue and directly act on cancer cells. **a** Localization of *CXCL13* − *CD8* + T cells and *CXCL13* + *CD8* + T cells in Patient 7. The regions enclosed by the yellow rectangle in the upper row are shown in the lower row. The green dashed lines are the tumour surface, and the yellow dashed lines are the contour lines at 30 µm intervals. **b** Image shows the tumour surfaces (green) and contours (red) at 30 µm intervals (−60 to 150 μm) in two areas in Patient 7. **c** Bar plot shows the proportions of CXCL13 + CD8 + T cells and CXCL13 − CD8 + T cells in each region. **d** *CXCL13* + *CD8* + T cells expressing *GZMB* were near or inside the tumour in Patients 1, 2, and 7.

Although the *CXCL13*+ cells we identified reportedly correlate with the therapeutic efficacy of anti-PD-L1 therapy, the localization and mechanism of these cells are unclear. We investigated this cellular function using spatial information and pathology-based analysis. In the cancer immune response, activated cytotoxic T lymphocytes (CTLs) directly contact and release perforin and granzymes, causing the cancer cell membrane to rupture and leading to apoptosis [36]. According to our pathology-based analysis, *CXCL13* + *CD8*+ cells directly engage with cancer cells, and most *CXCL13* − *CD8*+ cells were ubiquitously distributed in Patient 7 (Fig. 4a). We quantified the spatial localization of *CXCL13* + *CD8*+ and *CXCL13* − *CD8*+ cells inner-, peri-, and outer-region of the tumour (Fig. 4b, c). This trend of *CXCL13* + *CD8*+ cells engaging directly with cancer cells, as opposed to *CXCL13* − *CD8*+ cells, was further validated through region stratification analysis [37] across all examined fields. Furthermore, in all three post-PD-L1-CRT tissue samples, we confirmed that *CXCL13* + *CD8*+ lymphocytes expressed *GZMB* and directly interacted with cancer cells at the single-cell resolution. (Fig. 4d). Based on these results, we concluded that *CXCL13* + *CD8* + T cells are the main type of CTL that directly attacks cancer cells in the environment after treatment with anti-PD-L1-CRT.

### *CXCL13* + *CD8* + T cells are associated with the CXCL16-CXCR6 axis and linked to IFNG pathway activation

We identified distinct tumour areas in Patient 7 by HE staining; the presence of strong immune cell infiltration indicated active immune attack on cancer cells (“hot” pattern), and the absence of immune cells suggested reduced immune activation in surviving cancer cells (“cold” pattern). This dichotomy provides insights into the mechanisms of induced immune activation and evasion in the context of anti-PD-L1-CRT. Further analysis of this tissue was performed to unravel these complex immunological dynamics. We performed additional Xenium analyses of tissues from Patient 7 using a newly developed panel of 300 genes that specifically target the immune response, including DNA damage response genes (n = 19), nucleic acid sensor genes (n = 15), transcription factor genes (n = 21), chemokine/interleukin/interferon ligand receptor genes (n = 66), haematopoietic marker genes (n = 45), and immune checkpoint inhibitor target genes (n = 35) (Supplementary Table S2).

**Fig. 5:**
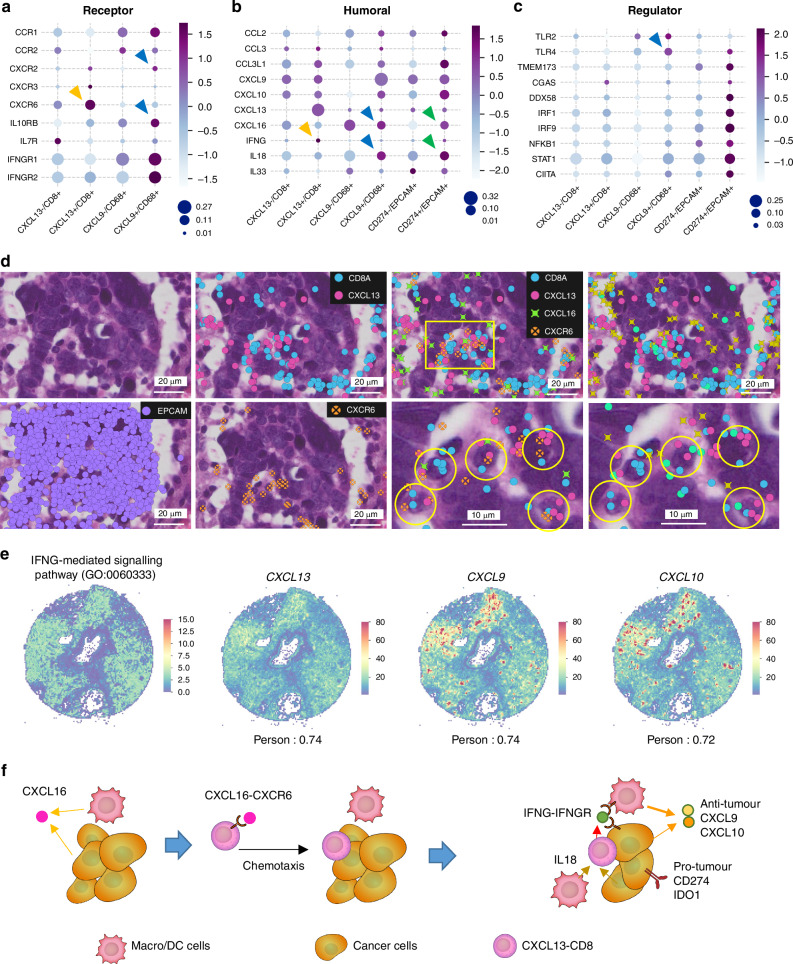
*CXCL13* + *CD8* + T cells accumulate via the *CXCL16*– *CXCR6* axis and trigger the IFNG pathway. **a** Bubble chart with a colour scale showing the average expression level and proportion of genes associated with receptors in *CXCL13* − *CD8* + , *CXCL13* + *CD8* + , *CXCL9* − *CD68* + , *CXCL9* + *CD68*+ and CXCL9 − CD68+ cells according to gene expression data for Patient 7. The x-axis indicates the cell type, the y-axis indicates the marker gene, the colour scale indicates the z score of the mean number of positive cells for each marker gene, and the circle size indicates the proportion of positive cells for each marker gene for each type of cell. **b, c** Bubble chart of genes associated with humoral immunity and regulators in *CXCL13* − *CD8* + , *CXCL13* + *CD8* + , *CXCL9*- *CD68* + , *CXCL9* + *CD68* + , *CXCL9* − *CD68* + , *CD274* − *EPCAM* + , and *CD274* + *EPCAM*+ cells. **d** *CXCL13* + *CD8* + T cells coexpress *IFNG*, and *CXCL9* and *CXCL10* expression is found near these cells. **e** Heatmaps indicating spatial enrichment of the IFN-mediated signalling pathway (log-adjusted P value) and expression levels of *CXCL13*, *CXCL9*, and *CXCL10* (normalized count). The values below the heatmaps are the Pearson correlation coefficients between expression levels and enrichment of IFN-mediated signalling pathways. **f** Models of cell‒cell interactions in the tumour environment.

Our initial focus was on understanding the mechanisms through which *CXCL9*+ cells and *CXCL13*+ cells are attracted to the tumour vicinity and evaluating chemokines, interleukins, and IFN receptors. We investigated expression of all of these receptors in tissues and found that *CXCR6* is a distinctive receptor on *CXCL13*+ cells by examining gene expression in these cells (Fig. 5a; Supplementary Fig. S6A). Expression of *CXCL16*, a ligand of *CXCR6* [38, 39], was expressed in *CD68*+ cells, including *CXCL9*+ cells, and partially induced in *EPCAM*+ cells (Fig. 4b and Supplementary Fig. S6B). *CXCR6* expression in *CXCL13* + *CD8*+ cells and *CXCL16* expression near *CXCL13*+ cells were visually confirmed by Xenium Explorer analysis (Fig. 5d and Supplementary Fig. S6D). In *CXCL9*+ cells, we identified distinctive candidate receptors, such as *CXCR2*, *IL10RB*, and *TLR4* (Fig. 5a-c and Supplementary Figs. S6A-C), which are involved in chemotaxis [40–42]; however, we could not confirm their association with specific ligands. We found that *CXCL9*+ cells produced *CCL18* (Supplementary Fig. S6B), a strong lymphocyte attractant, suggesting that *CXCL9*+ cells attract lymphocytes to the tumour vicinity.

Furthermore, we focused on the concordance between *CXCL13*+ cells and *IFNG*-expressing cells (Fig. 5b, orange arrow). We confirmed that *CXCL13* + *CD8*+ cells expressed *IFNG* (Fig. 5d), and we statistically verified the activation of *CXCL13* + *CD8*+ cells and the IFNG pathway (Fig. 5e). Furthermore, Xenium exploration confirmed that the majority of cells in the TME expressed *IFNGR* (Supplementary Fig. S6D). *CXCL9*, *CXCL10*, and *CD274* are reportedly downstream of *IFNG* [43]. We also statistically verified the correlation between the IFNG pathway and *CXCL9* and *CXCL10* expression (Fig. 5e). *IFNG* pathway total field analysis of *CXCL13* + , *CXCL9*+ and *CXCL10*+ cells using stLearn (v0.4.12) and GSEApy (v1.0.6) revealed correlation coefficients of 0.74, 0.74, and 0.72, respectively (Pearson, respectively). Thus, we created a hypothetical model of *CXCR6*-*CXCL16*-dependent chemotaxis of *CXCL13* + *CD8*+ cells and *IFNG*-dependent *CXCL9* and *CXCL10* expression (Fig. 5f).

### Characterization of hot and cold cancer cells after anti-PD-L1-CRT treatment

**Fig. 6:**
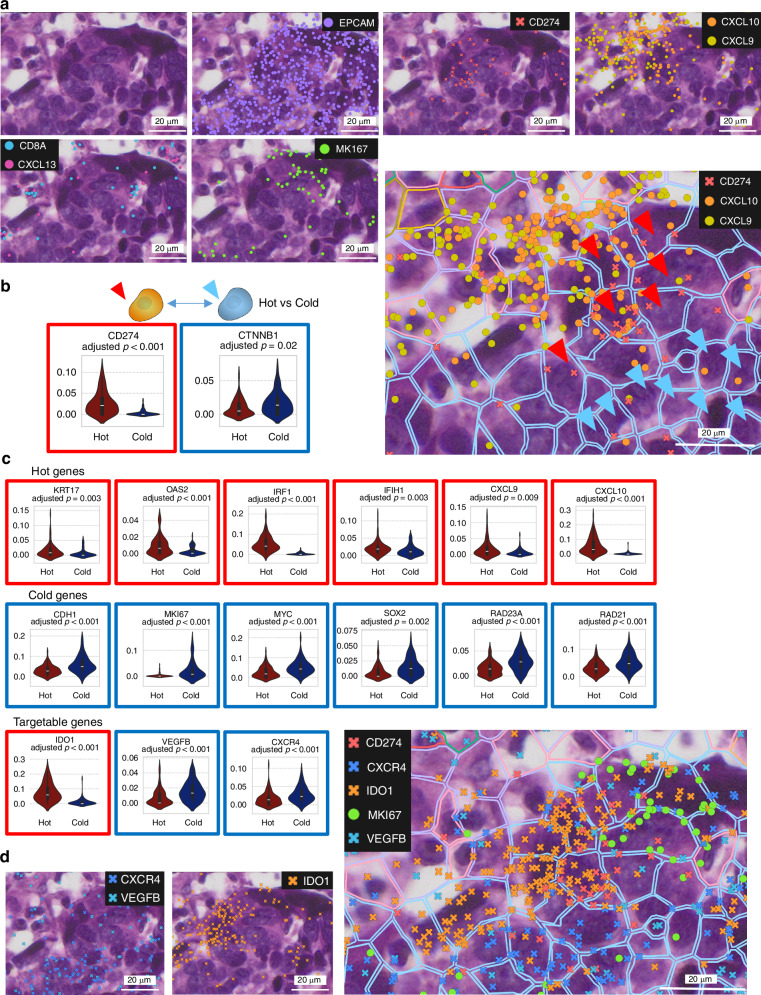
Characterization of immunologically hot and cold cancer cells after anti-PD-L1 combined with chemoradiotherapy. **a** Image of HE staining of mixed hot and cold tumours. **b**, **c** Violin plot of genes whose expression significantly differed (p < 0.05; Welch’s t test) between hot and cold tumours in Patient 7. The y-axis shows the density of gene expression per cell area (μm^2^). Genes are shown separately based on their categorization as hot, cold, or ICI-targetable genes. **d** Confirmation of gene expression through Xenium Explorer.

Our attention is now directed towards detailed investigation of the immune-cold state following anti-PD-L1-CRT, with subsequent comparative analysis against the immune-hot state. We examined gene expression within a representative one field of a pathological image that includes hot and cold region with gene expression profile, and we observed significant heterogeneity in *CD274* expression (Fig. 6a). Regions with high levels of *CD274* aligned with areas of high *CXCL9* and *CXCL10* expression. Conversely, in regions with low *CD274* expression, we detected *MKI67* expression, indicating high proliferative potential.

Given that states categorized as “cold” characterized by low immune cell infiltration coupled with high cellular proliferation can lead to treatment resistance, we conducted detailed gene expression analyses to identify features of immune-cold cancer cells. We also conducted both single-cell-based analyses (Fig. 6b-d) and region-based comparative analyses (Supplementary Fig. S7A) in the same field to identify features of immune-cold cancer cells. In single-cell analysis, we manually identified and precisely segmented hot and cold cancer cells within the regions with reference to the HE images. We defined *CD274* expression ≥ 2 within cells as immune-hot (Fig. 6a, red arrow) and 1 or 0 as immune-cold (Fig. 6a, blue arrow). Some cancer cells formed aggregates, and those with poor segmentation were excluded from the analysis. To mitigate the impact of segmentation errors during single-cell analysis, we concurrently conducted region-based gene expression analysis (Supplementary Fig. S7A). Welch’s *t* test was used for statistical analysis (Supplementary Table S3). Initially, we confirmed expression of *CD274* and *CTNNB1*, which are known markers of hot and cold regions, respectively [44].

In hot cells (100 cells), expression of genes such as *KRT17*, *OAS2*, *IRF1*, *IFIH1*, *CXCL9*, and *CXCL10* was significantly upregulated. In contrast, expression of such genes as *CDH1*, *MKI67*, *MYC*, *SOX2*, *RAD23*, *RAD21*, *HMGB1*, *VEGFB*, and *CXCR4* was significantly greater in cold cells (74 cells, Fig. 6c). Notably, a high proportion of the genes expressed in hot cells, such as *IFIH1, IRF1*, and *OAS2*, are related to the nucleic acid sensor pathway necessary for the RT-induced immune response [45–48]. In cold cells, expression of genes involved in cell proliferation, such as *MKI67* and *MYC*, as well as genes associated with DNA repair, including *SOX2*, *RAD21* and *RAD23* [39, 49–52], was increased. Additionally, to assess whether the features of the cold state are specific to post-PD-L1-CRT, we performed hot vs. cold single-cell analysis in Patient 12 post-CRT alone (Supplementary Fig. S7B). We observed a similar trend in single-cell analysis of gene expression in hot and cold cells isolated from tissue samples obtained from Patient 12. We identified potential therapeutic targets for both hot and cold cells, including *IDO1*, *VEGFB*, and *CXCR4*.

In this manner, even within the same cancer tissue, expression of *CD274* and hot/cold markers exhibited clear localization biases. Interestingly, hot and cold cell populations were observed to form a mosaic pattern, whereby clusters of several dozen hot or cold cells were observed within the same cancer cell mass (Supplementary Fig. S8). Attempts to convert cancer cells from cold to hot have attracted increased interest in cancer therapy. Therefore, this mosaicism represents a significant discovery, indicating that there are crucial factors (in addition to known factors such as the microbiome and driver genes) influencing hot/cold transitions.

## Discussion

This study utilized the single-cell spatial transcriptomics platform Xenium to explore immune environment alterations in NSCLC patient tissues following anti-PD-L1-CRT. One significant finding was that, even when immune cell levels decreased in peripheral blood, most immune cells within the tissue increased, and we identified distinct localized variations in immune activity. Moreover, we identified immune cells that directly act on cancer cells after treatment and elucidated their gene expression profiles and the surrounding immune environment. We identified *CXCL13* + *CD8* + T cells expressing *GMZB* and *IFNG*—cytotoxic T lymphocytes (CTLs)—as the cells that attack cancer cells following PD-L1-CRT. Previous studies have indicated that CXCL13, CXCL9, and CXCL10 are essential for the effectiveness of anti-PD-1/PD-L1 therapy [24–28] (without radiotherapy) and have been reported as potential biomarkers. Our findings elucidate that these cells directly engage cytotoxic T lymphocytes (CTLs) against cancer cells following PD-L1-CRT, suggesting their potential as biomarkers. Another important finding is that major pathological response (MPR), defined by the presence of less than 10% viable tumour cells, exhibited two distinct patterns of residual cancer cells. The residual tumour tissue could be classified into immunologically ‘hot’ and ‘cold’ regions. Notably, in certain ‘cold’ areas, we observed strong cellular proliferation, suggesting that immune suppression within these regions may contribute to continued tumour progression. These results, demonstrating that the residual tumour can be divided into immunologically ‘hot’ responsive regions and ‘cold’ resistant regions, underscore the importance of separately analysing these areas. Such an approach is critical for uncovering the mechanisms underlying immune-mediated tumour eradication and the immune evasion strategies that support tumour cell survival.

Subsequent analysis revealed that ‘immune-cold’ cells exhibited activated DNA repair mechanisms and enhanced proliferation. The cell-killing effects of radiation are induced through DNA damage, and radiation-induced immune responses are also triggered by DNA damage [48]. Therefore, increased DNA repair activity serves as a reasonable mechanism for both radiation resistance and suppression of CRT-induced immune responses. Furthermore, increased cellular proliferation was observed in cold cancer cells after treatment, suggesting their potential role as contributors to posttreatment recurrence and highlighting their role as crucial targets for new therapeutic strategies. Considering that DNA repair becomes activated in these cells, the efficacy of radiation dose escalation or additional DNA damage agents would be limited. Hence, our findings suggest the importance of exploring combinatory approaches involving cell proliferation pathways such as *CXCR4* and *VEGF*, which operate independently of DNA damage, to improve therapeutic outcomes.

On the other hand, in hot regions, *CXCL13* + *CD8* + T cells expressed exhaustion markers such as *TIGIT*, *LAG3*, and *CTLA4*. Since targeted therapies against these markers have been reported to further activate the immune response, they show promise as potential therapeutic targets to enhance the efficacy of PD-L1-CRT. Thus, our pathology-based single-cell spatial analysis is valuable, providing detailed insights into the TME alteration after PD-L1-CRT. This approach deepens our understanding of treatment responses and resistance. Notably, many of the genes identified in this study are confirmed clinical drug targets, such as anti-VEGF, PD-1/PD-L1 and anti-CTLA-4 antibodies, which have FDA approval. Also, antibodies targeting IDO1, LAG3 and TIGIT are in late stage clinical development and may be approved in the near future. Hence, our insights would contribute to future clinical trials and inform standard therapeutic approaches soon.

The limitation of this study is the sample size, as we analysed only five cases of PD-L1-CRT, and no prognostic analyses were conducted. Therefore, the current analysis primarily offers pathology-based biological insights, identifying potential marker candidates. The SQUAT trial involving 31 patients will allow for a correlation analysis with prognostic data once it becomes available, facilitating the validation of prognostic markers. Both anti-PD-L1 therapy and CRT act on nearly all solid tumours. Induction of *CXCL13*+ cells after ICI treatment has been reported in various solid cancers. Hence, the CXCL13–CD8-based immune mechanism proposed in our study may apply not only to ICI-CRT in NSCLC but also to other cancers and treatments.

In summary, single-cell spatial transcriptome analysis is a very effective tool for investigating the immune environment post-ICI-CRT at the single-cell level. Identification of *CXCL13* + *CD8* + -centred immune responses and immune-cold cancer cells after anti-PD-L1-CRT therapy holds promise for future developments in treatment strategies.

## Supplementary information

Supplementary Figures

Supplementary Tables

## Data Availability

The clinical specimen data, including Xenium data produced during this research, are archived in the DDBJ BioProject. The accession number is currently in the process of being requested. All the data necessary for assessing the conclusions presented in the paper can be found within the paper itself and/or in the Supplementary Materials.
