# Supplementary material for: Single-cell spatial analysis with Xenium reveals anti-tumour responses of CXCL13 + T and CXCL9+ cells after radiotherapy combined with anti-PD-L1 therapy
Source: Br J Cancer. 2025 Jul 16;133(6):795–808. doi: 10.1038/s41416-025-03088-0 (PMC12449477; doi:10.1038/s41416-025-03088-0)
Supplement: Supplementary file 1 — Supplementary Figures [file 41416_2025_3088_MOESM1_ESM.pdf]

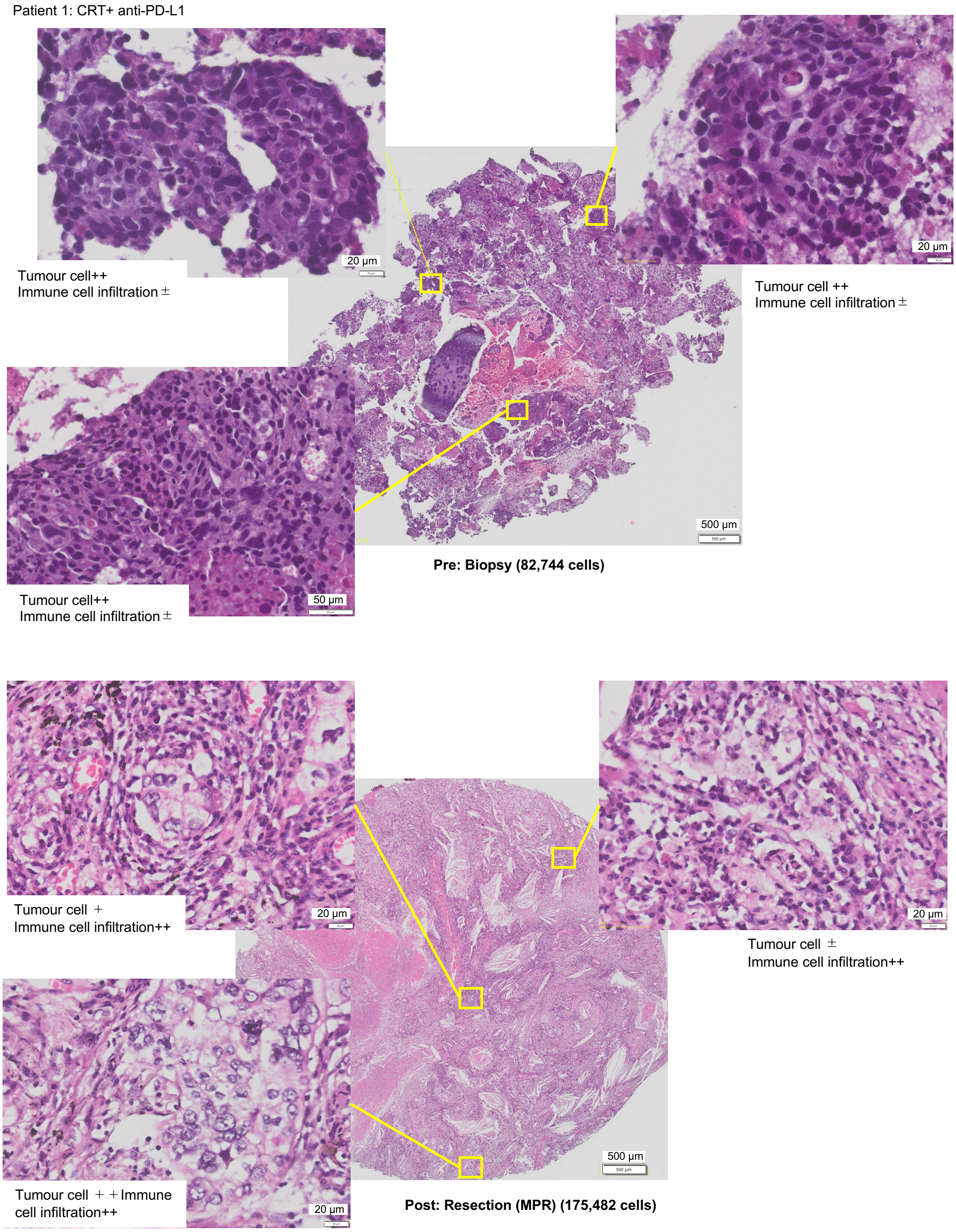

Figure S1A

HE staining and characteristics of each patient's tissue.

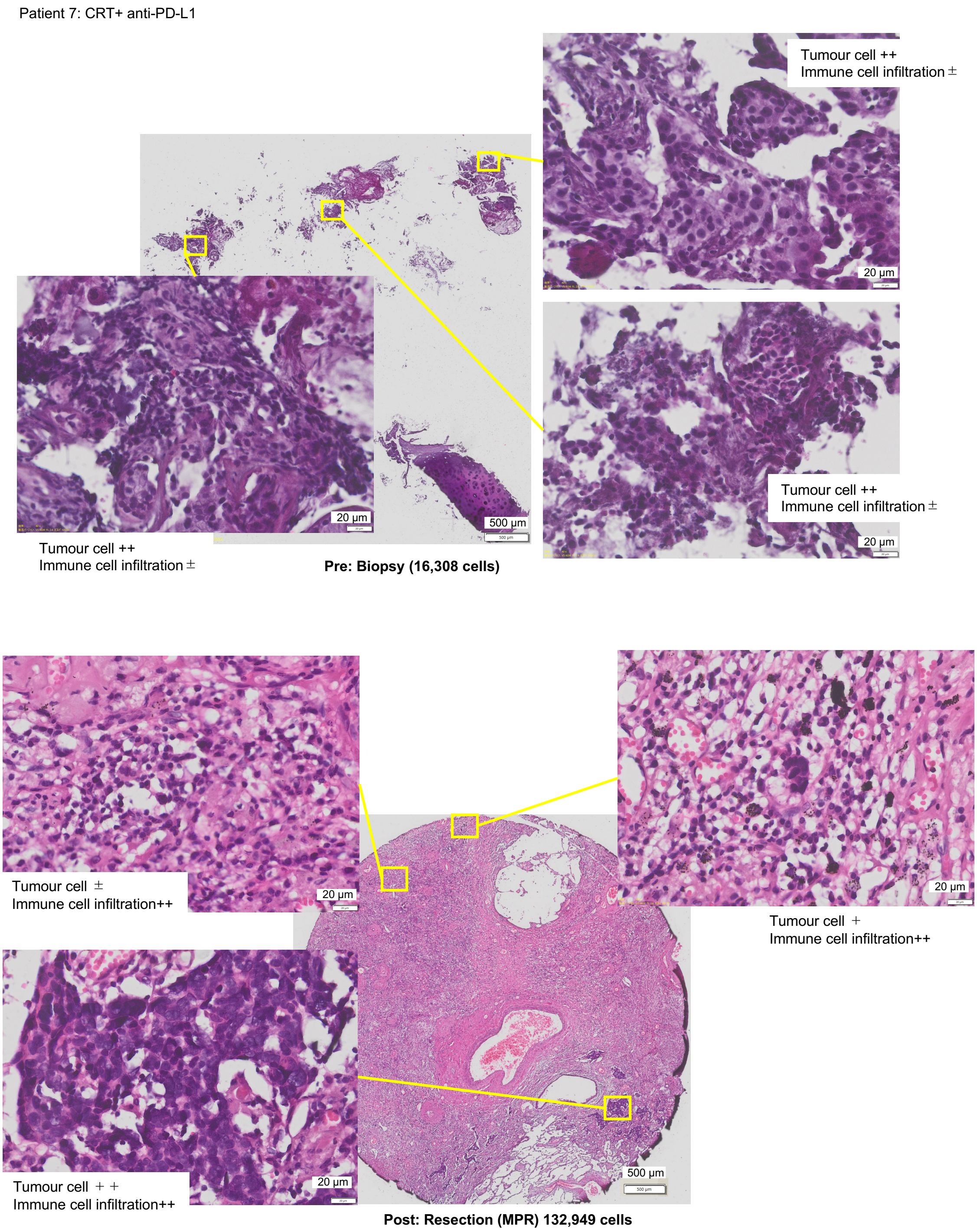

Figure S1B

HE staining and characteristics of each patient's tissue.

Patient 8: CRT+ anti-PD-L1

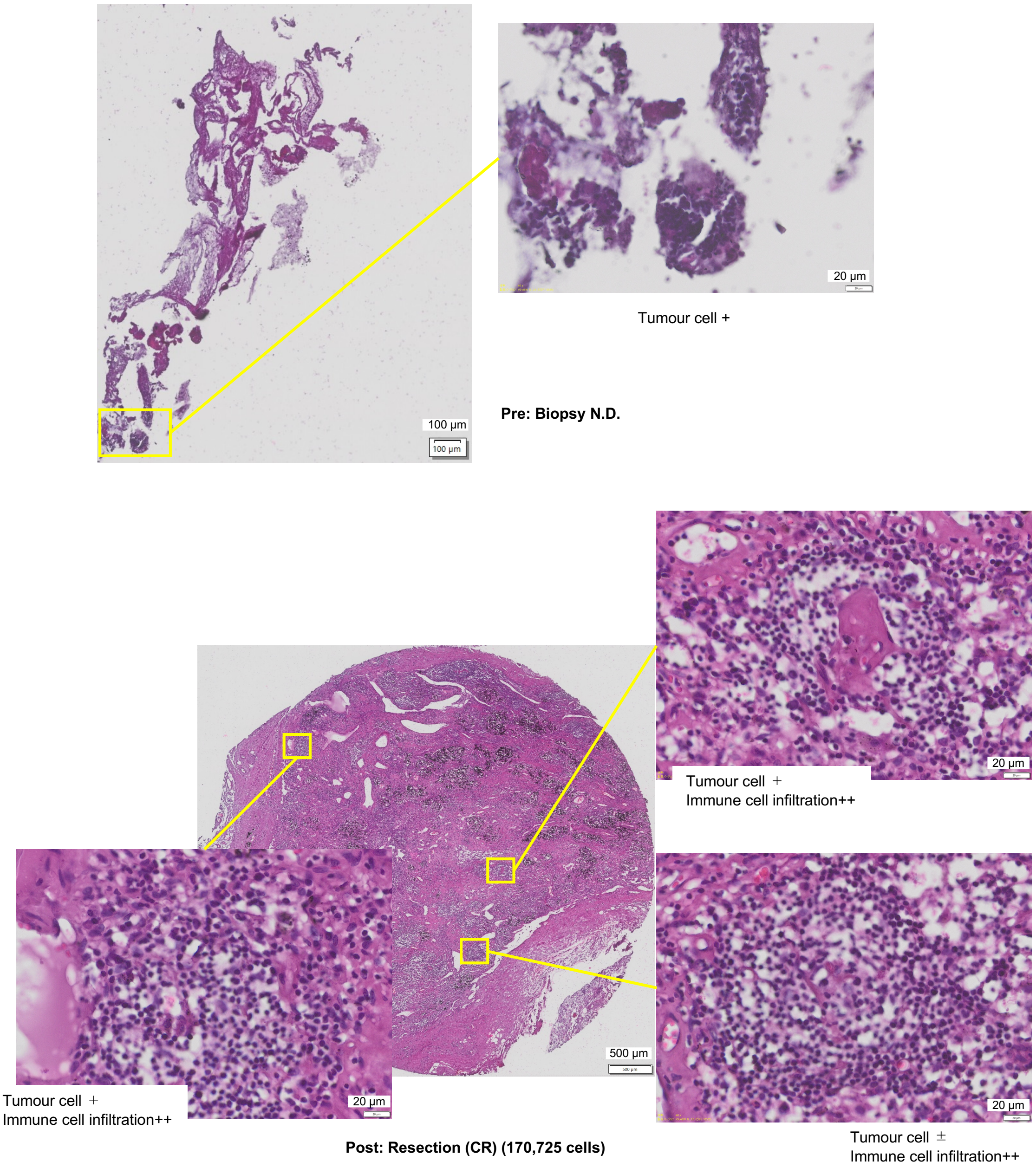

Figure S1C

HE staining and characteristics of each patient's tissue.

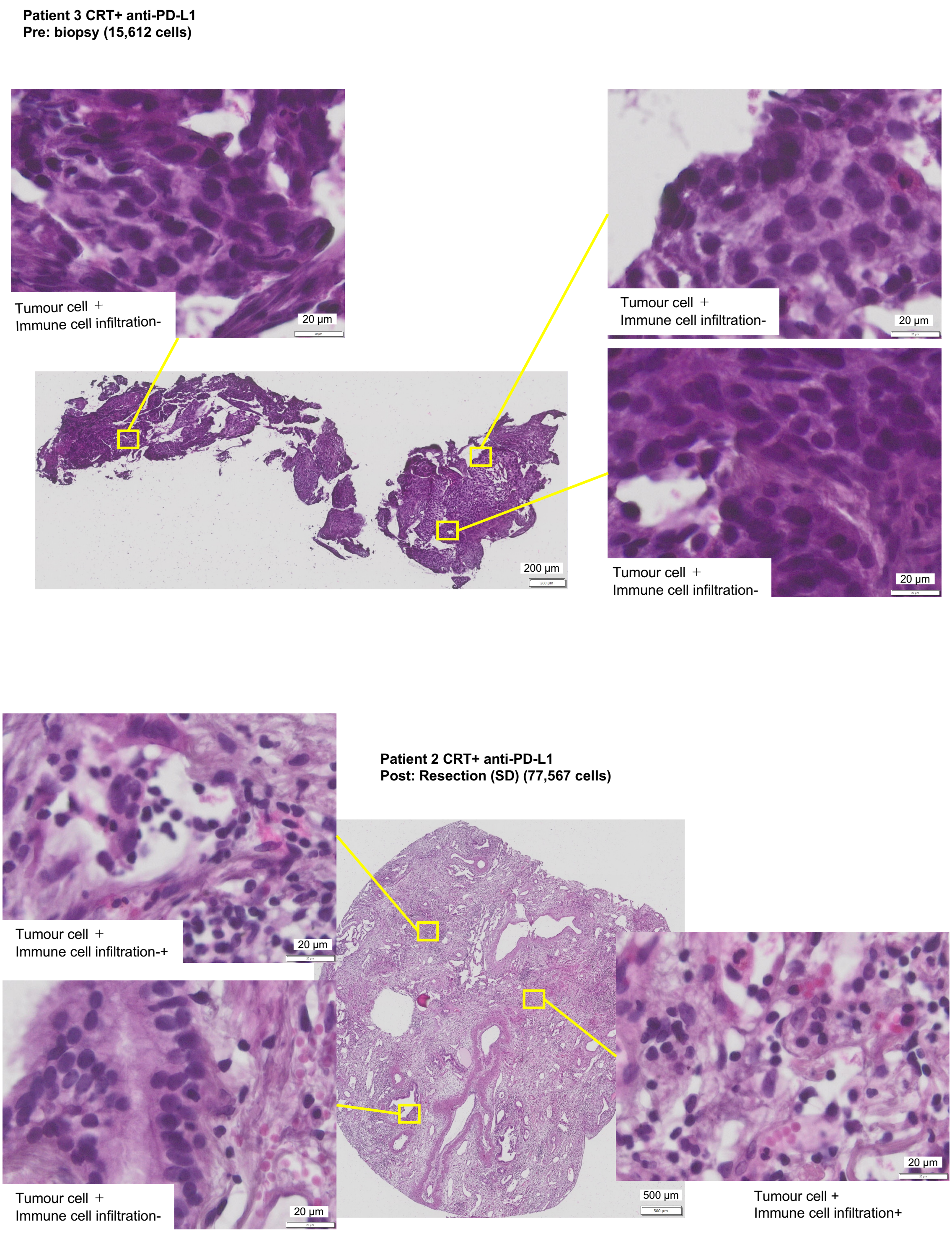

Figure S1D

HE staining and characteristics of each patient's tissue.

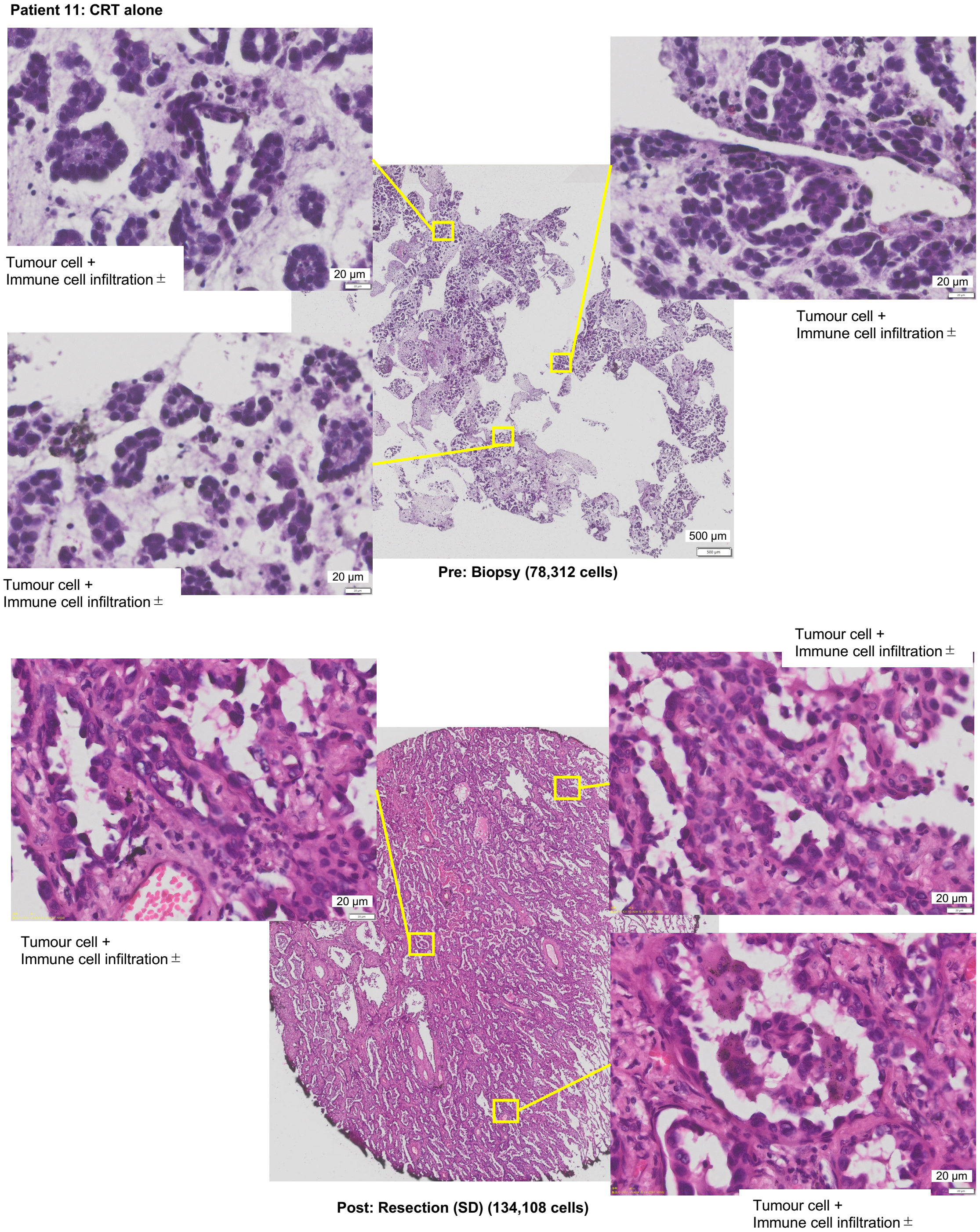

Figure S1E

HE staining and characteristics of each patient's tissue.

Patient 12: CRT alone

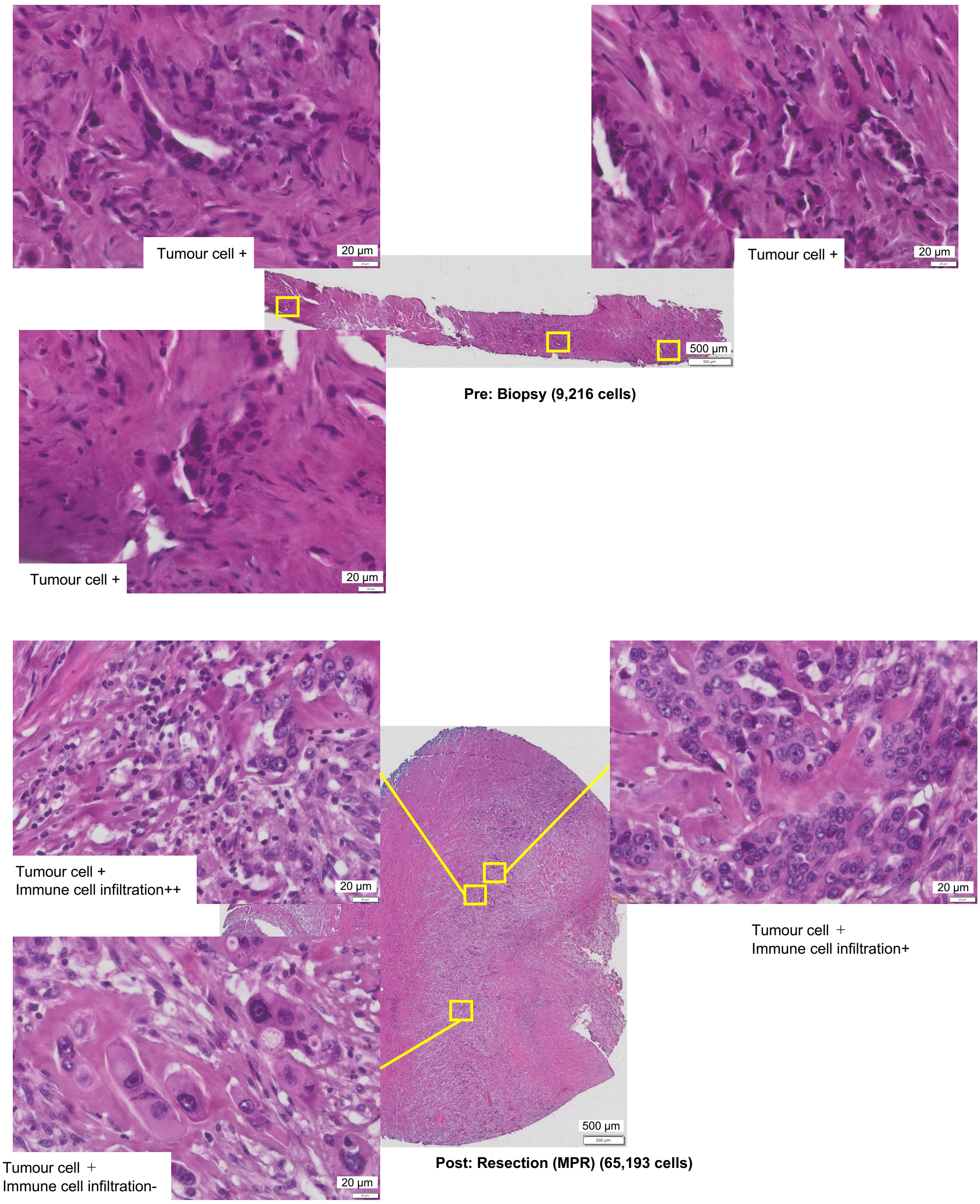

Figure S1F

HE staining and characteristics of each patient's tissue.

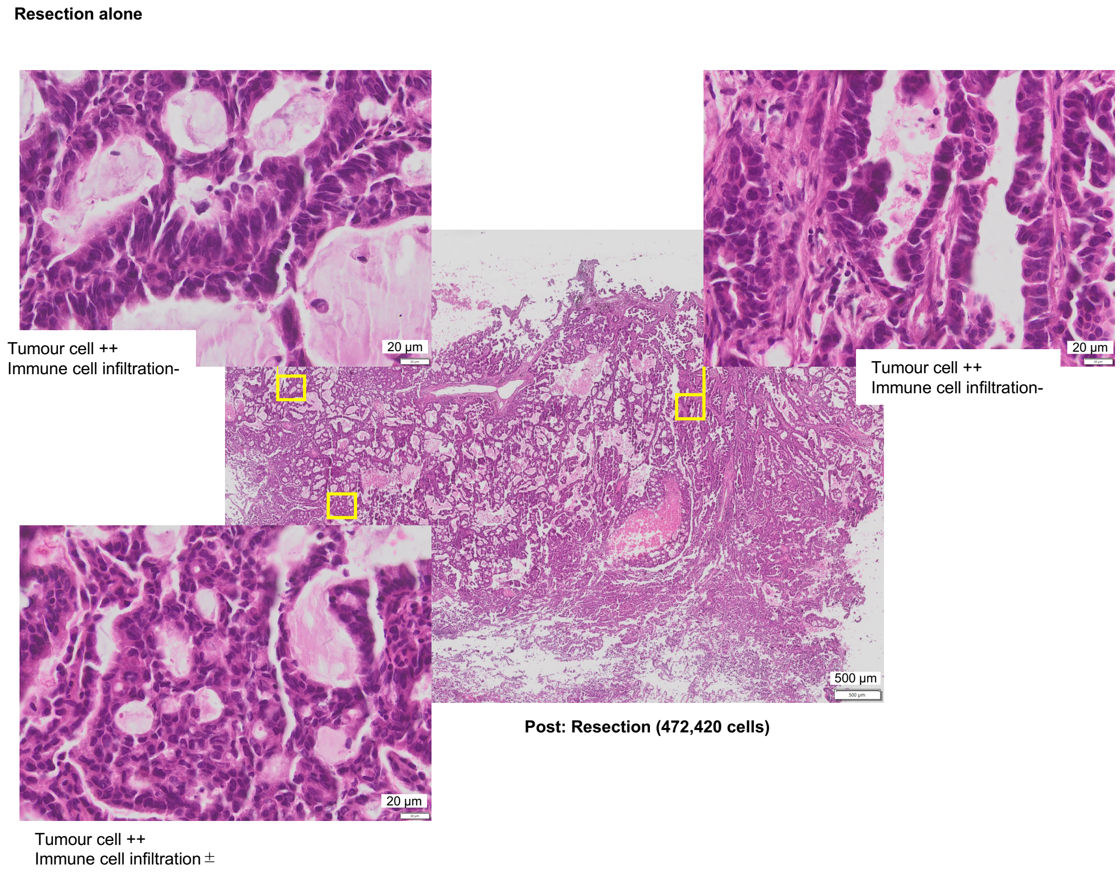

Figure S1G

HE staining and characteristics of each patient's tissue.

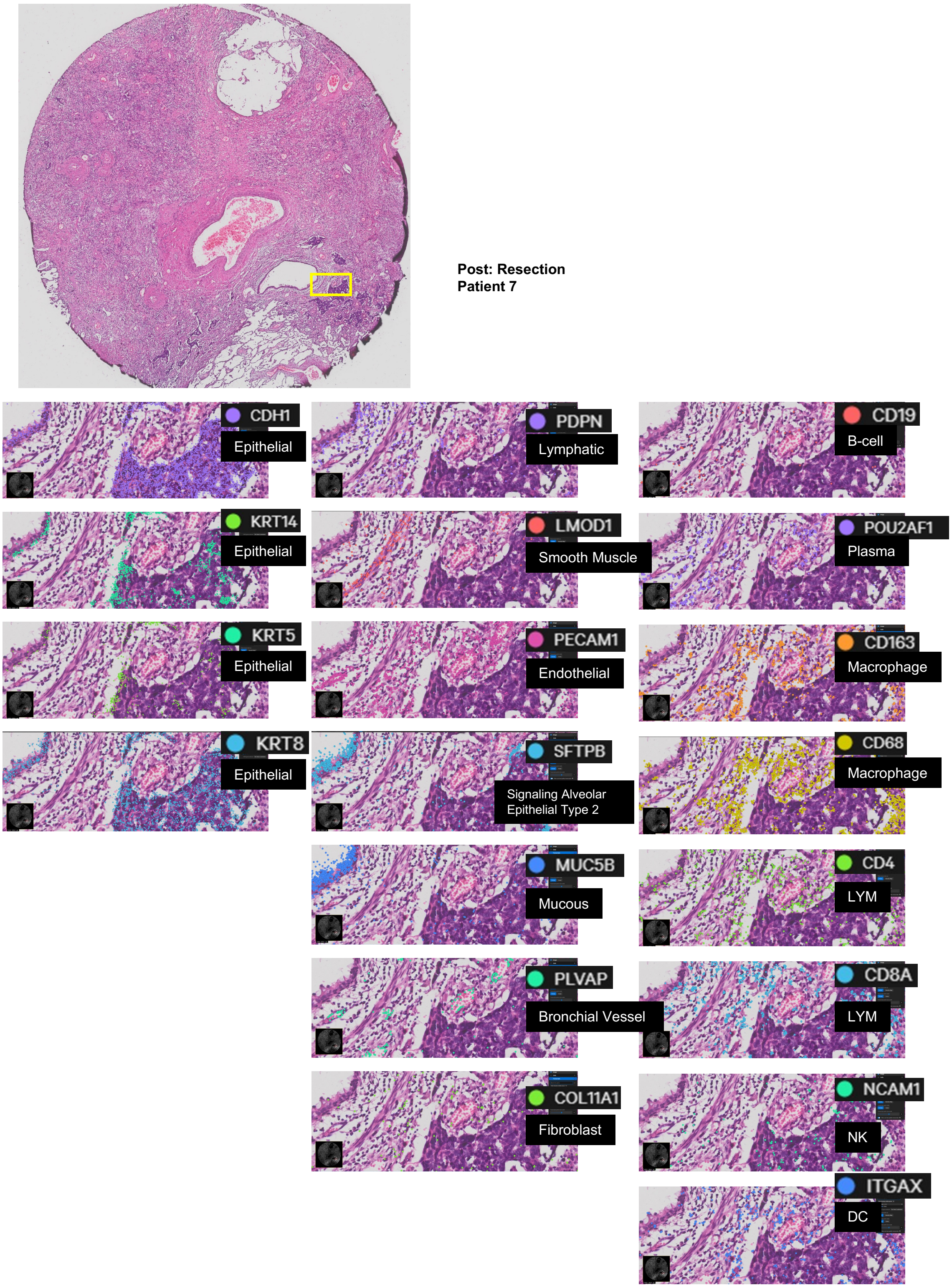

Figure S1H

Validation of Xenium through marker gene expression and HE stained tissue morphology.

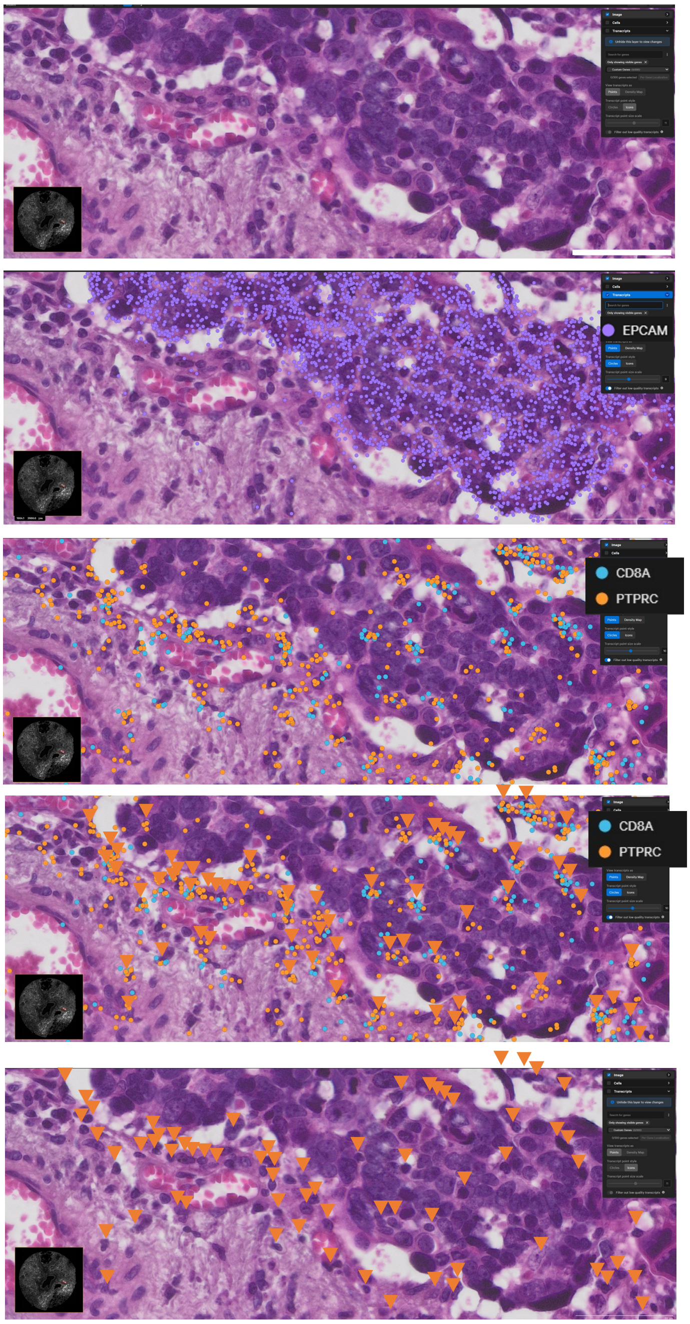

Figure S2

Identification of blood cells through the expression of blood cell markers (*PTPRC*, *CD8*). Evaluation of blood cell identification through HE staining revealed the best concordance with *PTPRC*+ cells.

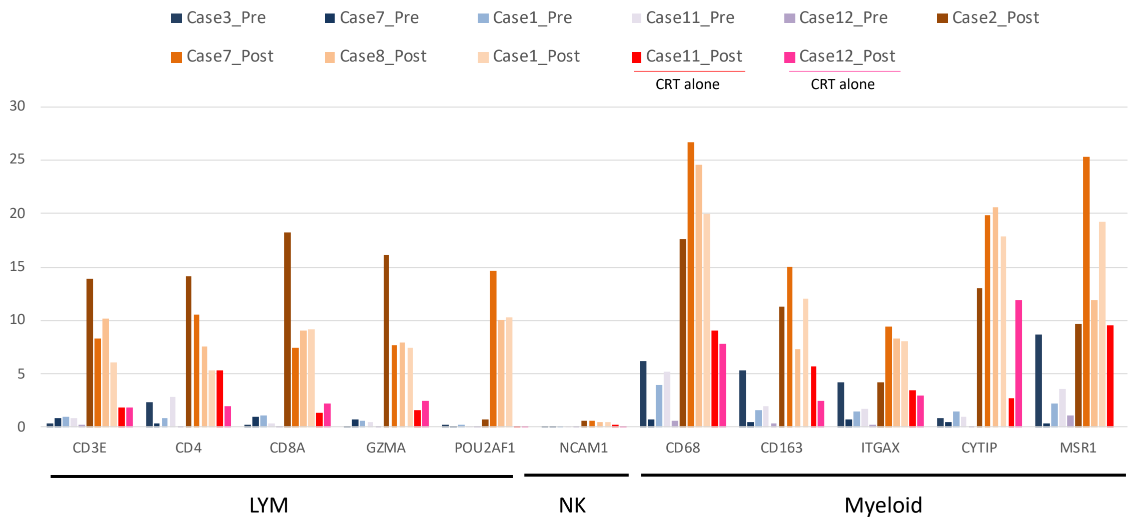

Figure S3 Comparison of immune cell in the tumor microenvironment among samples
The number of cells in the total field analysed before (Patients 1, 2, 7, 11, and 12) and after (Patients 1, 3, 7, 8, 11, and 12) surgery. Positivity was defined as ≥2 or above.

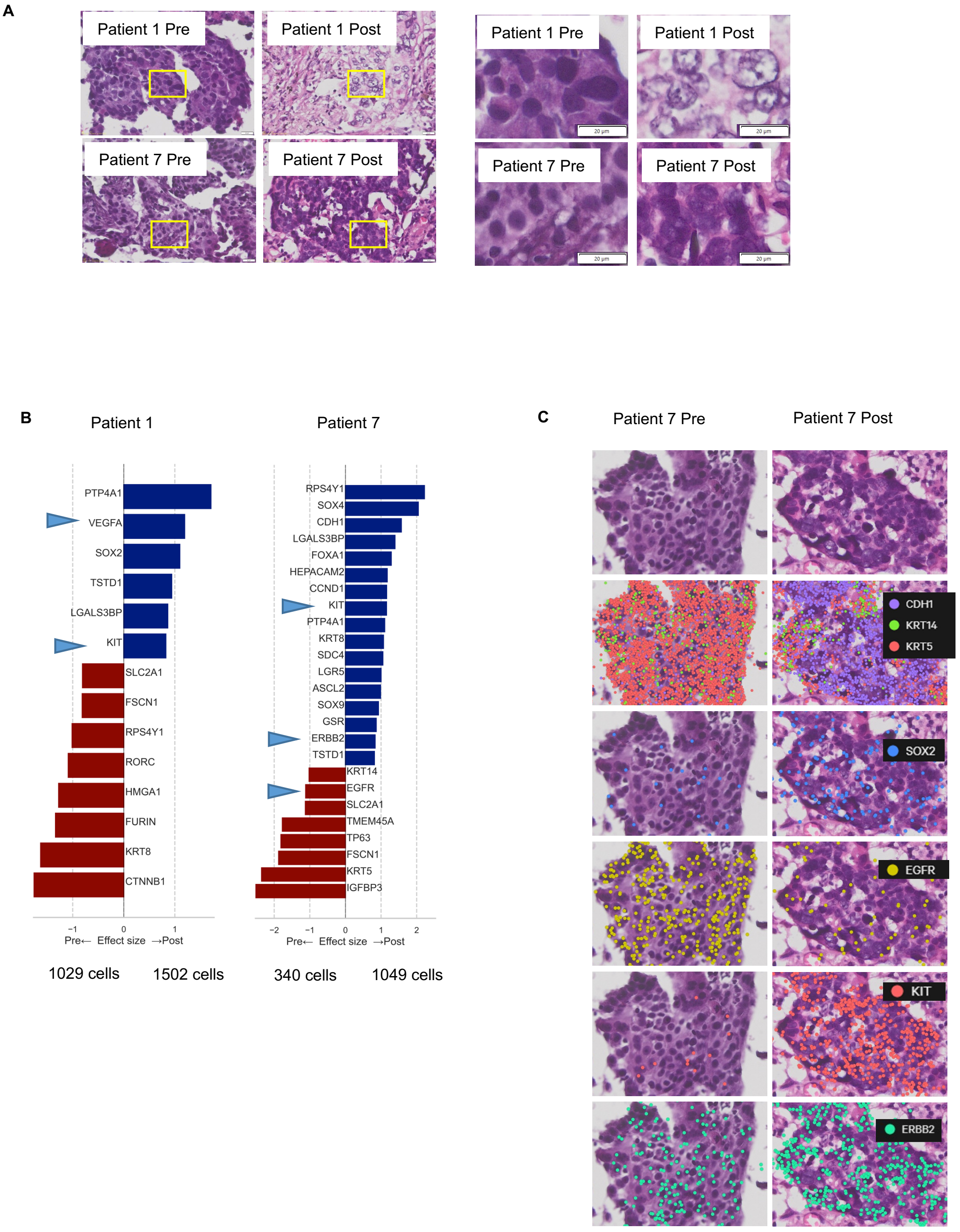

Figure S4

Characterization of cells and gene expression profile post anti-PD-L1-CRT in Patient 1 and Patient 7 tissue. **(A)** Confirmation of morphological changes before and after treatment in Patient 1 and Patient 7 by HE staining. **(B)** Investigation of gene expression changes in tumour cells in Patient 1 and Patient 7. **(C)** Confirmation of gene expression changes in Patient 7 by Xenium Explore.

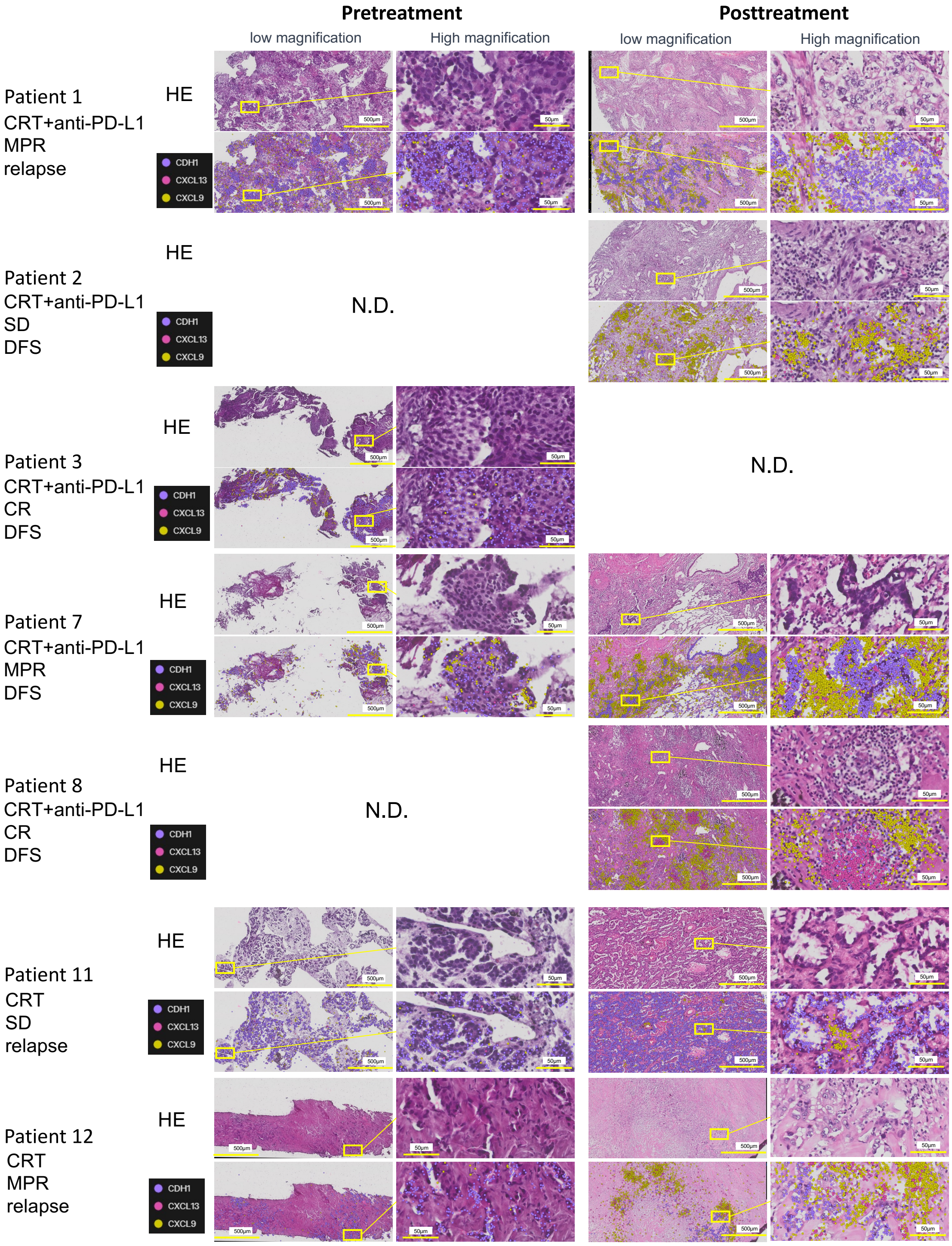

Figure S5

CXCL9 and *CXCL13* expression in pre- and post-treatment samples.

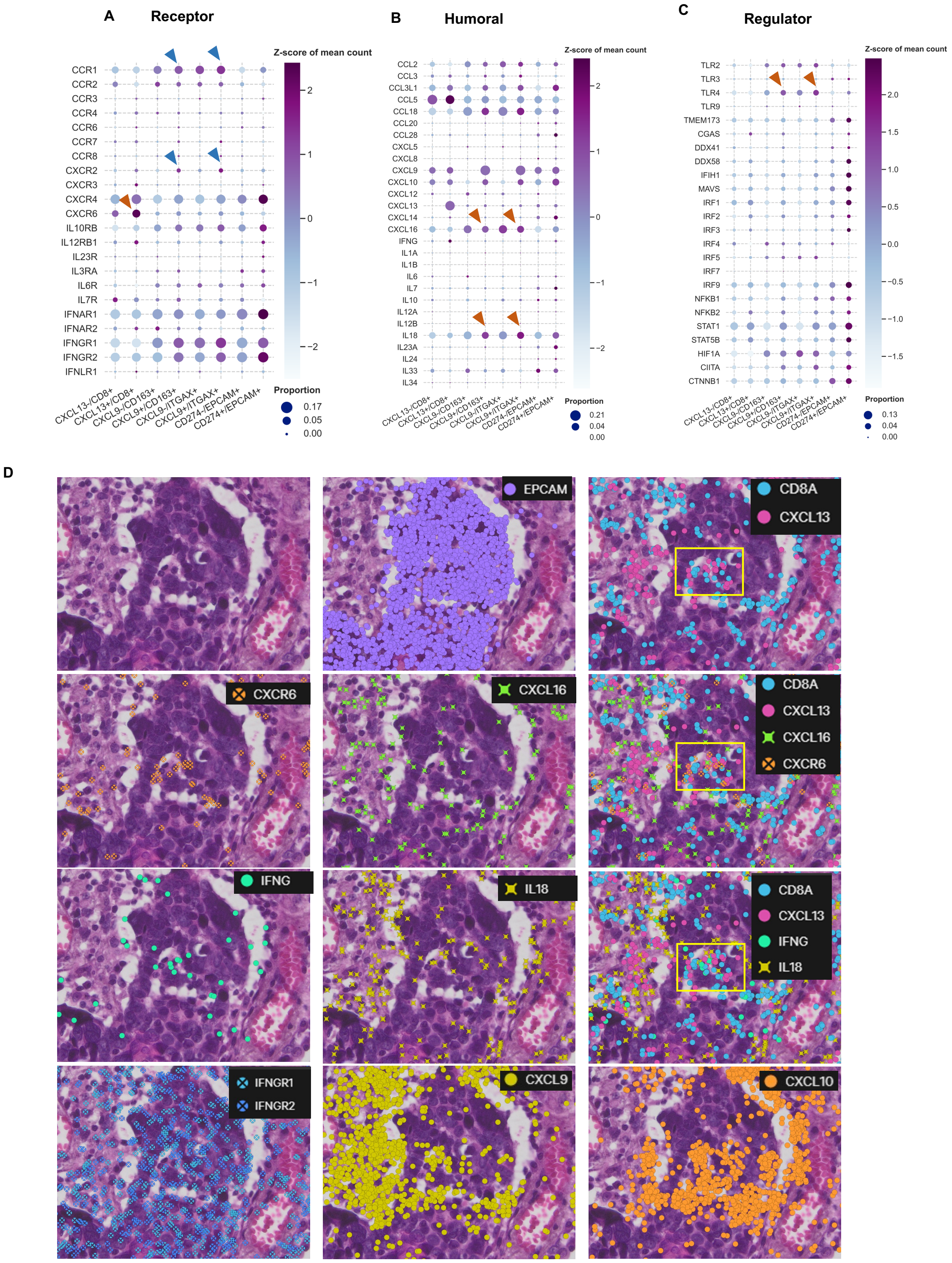

Figure S6

Screening of receptors, ILs/chemokines/IFNs, and regulators in *CXCL13*－ and *CXCL9*+ cells.

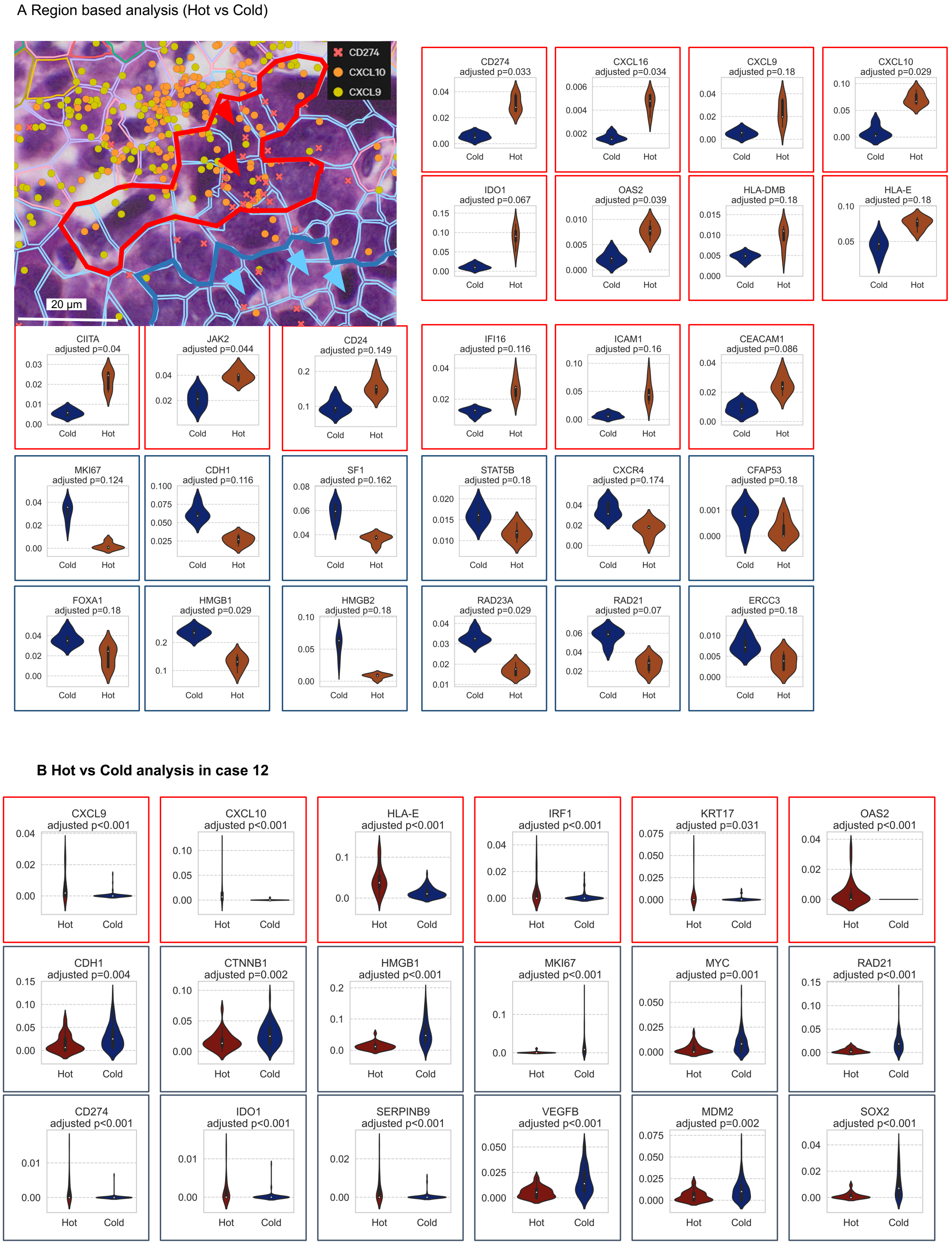

Figure S7

Characterization of immunologically hot and cold cancer cells after anti-PD-L1-CRT by **(A)** regional analysis in Patient 7 and **(B)** single-cell analysis in Patient 12

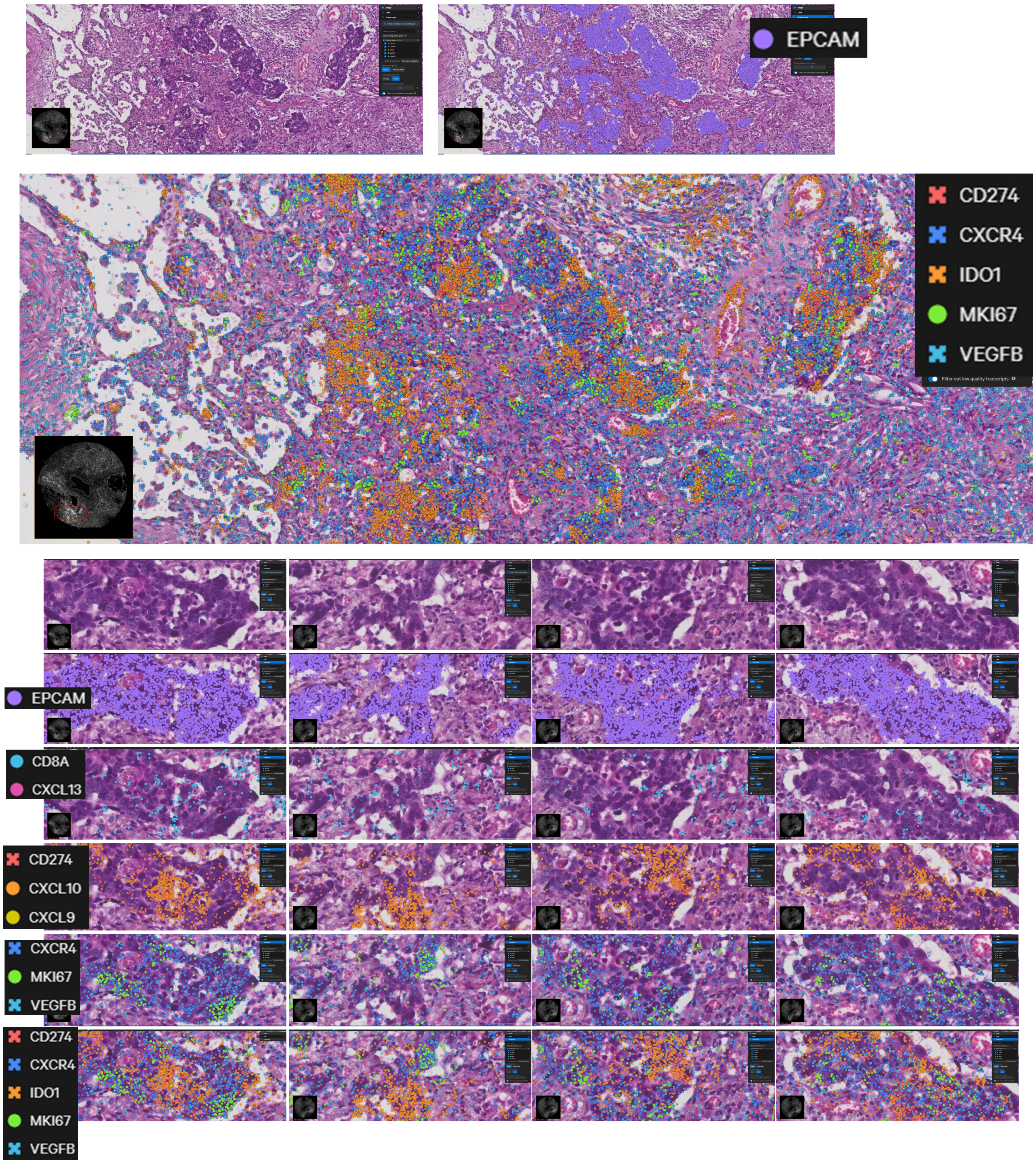

Figure S8

Posttreatment cancer cells form mosaic clusters expressing hot and cold marker genes.
